# Murine Functional Lung Imaging Using X-Ray Velocimetry for Longitudinal Noninvasive Quantitative Spatial Assessment of Pulmonary Airflow

**DOI:** 10.3390/tomography11100112

**Published:** 2025-10-02

**Authors:** Kevin A. Heist, Christopher A. Bonham, Youngsoon Jang, Ingrid L. Bergin, Amanda Welton, David Karnak, Charles A. Hatt, Matthew Cooper, Wilson Teng, William D. Hardie, Thomas L. Chenevert, Brian D. Ross

**Affiliations:** 1 Department of Radiology Center for Molecular Imaging, University of Michigan School of Medicine, Ann Arbor, MI 48109, USA; heistka@umich.edu (K.A.H.); bonhachr@umich.edu (C.A.B.); jyoungso@umich.edu (Y.J.); awelton@umich.edu (A.W.); tlchenev@umich.edu (T.L.C.); 2 Unit for Laboratory Animal Medicine, University of Michigan School of Medicine, Ann Arbor, MI 48109, USA; ingridbe@umich.edu; 3 Department of Radiation Oncology, University of Michigan School of Medicine, Ann Arbor, MI 48109, USA; karnakd@umich.edu; 4 4DMedical Research and Development, Melbourne, VIC 3053, Australia; chatt@4dmedical.com (C.A.H.); mcooper@4dx.com (M.C.); wteng@4dmedical.com (W.T.); 5 Department of Pediatrics, Division of Pulmonary Medicine, Cincinnati Children’s Hospital Medical Center, University of Cincinnati, Cincinnati, OH 45229, USA; william.hardie@cchmc.org; 6 Department of Biological Chemistry, University of Michigan School of Medicine, Ann Arbor, MI 48109, USA

**Keywords:** functional pulmonary imaging, four-dimensional X-ray velocimetry (4DXV), mouse lung imaging, regional airflow, pulmonary fibrosis

## Abstract

**Background/Objectives:** The recent development of four-dimensional X-ray velocimetry (4DXV) technology (three-dimensional space and time) provides a unique opportunity to obtain preclinical quantitative functional lung images. Only single-scan measurements in non-survival studies have been obtained to date; thus, methodologies enabling animal survival for repeated imaging to be accomplished over weeks or months from the same animal would establish new opportunities for the assessment of pathophysiology drivers and treatment response in advanced preclinical drug-screening efforts. **Methods:** An anesthesia protocol developed for animal recovery to allow for repetitive, longitudinal scanning of individual animals over time. Test–retest imaging scans from the lungs of healthy mice were performed over 8 weeks to assess the repeatability of scanner-derived quantitative imaging metrics and variability. **Results:** Using a murine model of fibroproliferative lung disease, this longitudinal scanning approach captured heterogeneous progressive changes in pulmonary function, enabling the visualization and quantitative measurement of averaged whole lung metrics and spatial/regional change. Radiation dosimetry studies evaluated the effects of imaging acquisition protocols on X-ray dosage to further adapt protocols for the minimization of radiation exposure during repeat imaging sessions using these newly developed image acquisition protocols. **Conclusions:** Overall, we have demonstrated that the 4DXV advanced imaging scanner allows for repeat measurements from the same animal over time to enable the high-resolution, noninvasive mapping of quantitative lung airflow dysfunction in mouse models with heterogeneous pulmonary disease. The animal anesthesia and image acquisition protocols described will serve as the foundation on which further applications of the 4DXV technology can be used to study a diverse array of murine pulmonary disease models. Together, 4DXV provides a novel and significant advancement for the longitudinal, noninvasive interrogation of pulmonary disease to assess spatial/regional disease initiation, progression, and response to therapeutic interventions.

## 1. Introduction

Preclinical pulmonary disease models are commonly used to provide biological and functional insights into mechanisms associated with disease processes, progression, and the effectiveness of therapeutic intervention. Global lung function and tissue/airway mechanics are common measurements evaluated using forced oscillation techniques such as those provided by the Scireq flexiVent^®^ system [1,2]. This technology enables the assessment of respiratory compliance and resistance, tissue damping and elastance, central airway resistance, forced vital capacity (FVC), forced expired volume (FEV), and forced expired flow (FEF). The visualization of lung structure and function has also been significantly advanced by pulmonary CT and MRI scans [3,4,5,6,7]. CT is widely applied clinically for the assessment of air trapping, emphysema, and bronchiectasis, with recent advancements in image processing having enabled the visualization of small airway disease [8], while advanced MRI techniques have used inhaled hyperpolarized gases to probe lung structure and function [9,10,11,12]. Each available pulmonary image-based measurement has important roles in the preclinical and clinical evaluation of lung function and disease phenotypes [13]. Moreover, contrast-enhanced CT and MRI imaging can enable ventilation and perfusion imaging of the lung at a single point in time but cannot be used to measure time-varying respiratory dynamics.

**Figure 1:**
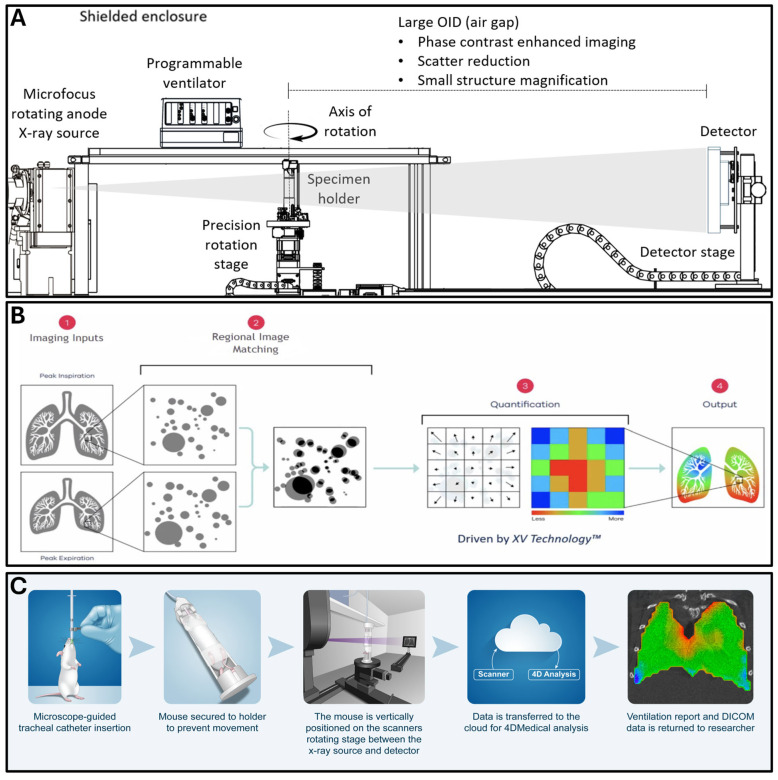
(**A**) The 4DXV instrument hardware is depicted, which consists of an X-ray source, a detector, and a specimen holder which secures an animal in the X-ray beam while rotating on a stage with precision angular rotation in tandem with a programmable ventilator. This system is designed to provide phase contrast lung images of mice. (**B**) 1. X-ray imaging datasets are obtained by the scanner during ventilation to derive peak inspiration and peak expiration images for computational analysis. 2. Regional image matching software evaluates regional changes between ventilation phases for a single animal. 3. Quantitation of changes is performed by 4DMedical software algorithms. 4. Heat map overlays are computationally derived, depicting quantitative regional changes in functional metrics within the lung parenchyma. (**C**) The animal preparation process for imaging includes anesthesia, tracheal catheter insertion, and the securing of the animal to the specimen holder for placement between the X-ray source and the detector. The vertically positioned animal undergoes X-ray scanning, and data is transmitted to the cloud for analysis by 4DMedical software algorithms. Data including ventilation reports and DICOM files are returned to researchers for evaluation. Danielle Dobbs assisted with the preparation of the artistic drawings using Adobe Illustrator (version 29.3; www.adobe.com, accessed on 17 June 2025).

A recent development in functional lung imaging, X-ray velocimetry (XV), allows for the visualization and quantitation of lung tissue movement at exquisite spatial and temporal resolution, enabling cinematic pulmonary ventilation measurements (Figure 1A) [14,15]. XV provides a three-dimensional, time-resolved ventilation image of the lung allowing for the study of time-varying respiratory dynamics. XV measures time-varying ventilation dynamics from lung images using a volumetric particle image velocimetry (4DPIV) image-processing algorithm, which tracks local lung tissue movement, allowing for a high-spatial-resolution measurement of lung tissue expansion (i.e., local changes in lung volume, Figure 1B). This information is used to generate regional ventilation maps for airway visualization and analysis. The identification of ventilation–perfusion mismatch at high speed and high resolution is achieved, allowing for quantitative, image-derived outputs for the visualization of compromised ventilation and assessment of pulmonary disease heterogeneity. The XV approach has recently been used to evaluate murine models of asthma [16] and muco-obstructive lung disease [17], and these initial studies have demonstrated the power and unique ability of XV technology to detect, quantify, and spatially visualize varying pathological changes in murine models of lung disease. In a recent rat study, the effects of localized physical obstruction were evaluated following agar bead delivery to the lung, which resulted in an alteration in ventilation parameters [18]. Similarly, two rat cystic fibrosis models were also scanned by XV imaging, which demonstrated that altered respiratory mechanics could be visualized and quantified [18].

The capacity to use quantitative imaging readouts for the detection of early disease initiation and rapid evaluation of experimental drugs is critical for the effective screening of therapeutic efficacy. There is a clear need for the development of advanced physiological methods that allow for nonsurgical XV longitudinal imaging studies where repeated measurements from individual animals can be obtained at multiple time intervals to follow biological progression or responses to therapy. Previous XV scanning studies have used a non-survivable, tracheostomized animal preparation connected to the scanner’s ventilation system to provide respiratory support. In the present study, we developed anesthesia management protocols that enable full and rapid recovery, permitting the acquisition of multiple longitudinal XV scans from a single animal for the assessment of pulmonary changes over time. Test–retest scanning on normal mice was performed over 8 weeks to evaluate the repeatability of quantifiable metrics derived from the scanner. Procedures developed for longitudinal scanning were applied to monitor the disease progression of a genetically engineered pulmonary fibroproliferative disease model to demonstrate the potential for the investigation of lung physiology and pulmonary function through repeat scanning. Using X-ray dosimetry studies to assess and quantify the radiation dose received from a single-scan procedure, we modified the image acquisition parameters to minimize X-ray exposure, providing further opportunities for repeat scanning protocols by reducing concern regarding cumulative radiation effects on underlying pulmonary biology. Overall, the aim of this study was to develop and evaluate longitudinal XV scanning protocols for repeat measurements of animal cohorts to support routine assessment of functional lung changes across disease models, to facilitate translational advances in lung pathophysiology and clinical care.

## 2. Methods

### 2.1. Reversible Anesthesia and Tracheal Intubation Procedure

All animal procedures were approved by the University of Michigan Institutional Animal Care and Use Committee and were performed in compliance with all relevant ethical regulations therein, including the ARRIVE guidelines (www.arriveguidelines.org). All rodents were maintained in a specific pathogen-free barrier unit at the University of Michigan accredited by the Association for Assessment and Accreditation of Laboratory Animal Care (protocol number PRO00011252, approval date 31 January 2023). Mice were administered intraperitoneally with a mixture of 23 mg/mL ketamine (MWI, Boise, ID, USA) and 0.4 mg/mL dexmedetomidine (Zoetis Inc., Kalamazoo, MI, USA) to deliver a combination dose of 76.7 mg/kg ketamine and 1.3 mg/kg dexmedetomidine. The depth of anesthesia was verified by toe pinch prior to intubation. Mice were positioned vertically on a specially designed holder for intubation with upper incisors held by a horizontally fastened length of suture. With the tongue placed gently to one side, the vocal cords were easily visualized through the microscope. A calcium hydroxide placement tool was used to briefly touch the vocal cords and open the airway for intubation. Once inserted, the guide tool was disconnected from the intubation tube, and the normal breath rate was verified. A piece of 3M Transpore tape (3M, Saint Paul, MN, USA) was used to secure the intubation tube to ensure stability during imaging (Figure 1C). During imaging, 2% isoflurane was supplemented through the ventilator to facilitate compliance and provide an adjustable measure of anesthetic depth during the scanning procedure. The vertically positioned mouse was secured inside the scanner to a turntable between the X-ray source and the detector for initiation of the scanning procedure (Figure 1C). The dexmedetomidine sedation was reversed using Atipamezole hydrochloride (https://www.tocris.com) prepared by the dissolution of 10 mg in 25 mL of 0.9% NaCl, USP, to make a concentration of 0.4 mg/mL. Each mouse was administered 100 μL via subcutaneous injection, immediately after removal from the ventilator. Following completion of the scan, the imaging data was transmitted to the cloud for analysis by 4DMedical Inc., the manufacturer of the XV scanner used in the current studies. Engineers at 4DMedical processed the acquired imaging data to generate ventilation reports and produce digital imaging and communications in medicine (DICOM) files, which were electronically transmitted back to the researchers for evaluation and interpretation (Figure 1C).

### 2.2. Dynamic Functional Lung Imaging

Mice were imaged on a 4DMedical Permetium imaging system with XV Technology (Notting Hill Devices, Melbourne, Australia). This system utilizes unique X-ray velocimetry technology from 4DMedical to quantify regional changes in lung function, allowing for dynamic characterization of lung kinetics. Mice were secured to an upright bed on a rotating stage located between the X-ray source and detector (Figure 1A,C). The stage includes rotation as well as x, y, and z-plane controllers, allowing for the precise positioning of animals of various ages and body weights. The scanning parameters used for the fibroproliferative disease model studies included a scan rate of 800 projections per time point (PPTP), 176 breaths per minute, an X-ray filter of 32 μm Mo, and a scan time of 4 min 38 s. Alternate scan parameters were additionally explored, including variations in breath rates, X-ray filter thickness, and scan rates, to examine the ability to reduce lung X-ray exposure while maintaining the ability to derive accurate quantitative imaging scan metrics.

### 2.3. Radiation Dosimetry Measurement

Three layers of approximately 10 × 2.5 × 0.4 cm pieces of solid water material [19] were secured in the beam path. Gafchromic films (Ashland Inc., Wilmington, DE, USA) were affixed in front, between the first and second layer, and behind the solid water in relation to the beam focal spot before the stage began its movement for the scan, and analyzed to determine the accumulated radiation dose as described previously [20]. Briefly, experimental duplicates of irradiated film images were digitized, and analyzed using commercial proprietary software (FilmQA Pro 2026 v 5.0, Ashland Inc.). The calibration procedure involves corrections for both non-linear responses of optical density to dose and film/scanner non-uniformities. Several additional dosimetry experiments were undertaken to further quantify and evaluate the impact of varying scan acquisition parameters on the accumulated radiation dose and functional lung metrics derived from image processing.

### 2.4. Fibroproliferative Lung Mouse Model

We used CCSP-rtTA^+/−^ activator mice (CCSP/-) expressing the reverse tetracycline-responsive transactivator (rtTA) fusion protein under the control of the rat Clara Cell Secretory Protein (CCSP) gene promoter [21] and (TetO)7-cmv TGFα transgenic mice, which are conditional doxycycline (Dox)-inducible transgenic mice containing the human TGFα cDNA under the control of seven copies of the tetracycline operon plus a minimal CMV promoter [22]. To produce single transgene (CCSP-rtTA^+/−^) and bitransgenic (CCSP-rtTA^+/−^/(TetO)7-cmv TGFα^+/−^) mice, homozygous CCSP-rtTA^+/+^ activator mice were mated to hemizygous (TetO)7-cmv TGFα^+/−^ mice (CCSP/TGFα). All mice were derived from the FVB/NJ inbred strain. To induce TGFα expression, doxycycline (Sigma, St. Louis, MO, USA) was administered in the drinking water at a final concentration of 0.5 mg/mL with 1% ethanol along with dosing in food at 625 mg/kg. The doxycycline water was replaced three times per week. A total of CCSP/- (*n* = 9) and CCSP/TGFα (*n* = 15) were prepared for these studies.

### 2.5. Lung Inflation with Vascular Perfusion–Fixation

Tissue preparation and fixation were performed as previously described [23,24]. Briefly, mice were surgically prepared to allow for lung inflation and perfusion–fixation into the right ventricle of the heart. Lungs were fixed overnight, followed by processing for histological staining and evaluation.

### 2.6. Histological Evaluation

Histological sections were evaluated using light microscopy at magnifications ranging from 20× to 600× by a board-certified veterinary pathologist while blinded to the study groups. Lesions were assessed descriptively and by quantitative analysis according to the International Harmonization of Nomenclature and Diagnostic Criteria (INHAND) consensus guidelines for rodent toxicologic pathology [25]. Lesion severity was scored as 0: not present; 1 (mild): focal or low-density multifocal; 2 (moderate): locally extensive or higher-density multifocal; 3 (severe): regionally extensive or high-density coalescing; and 4 (marked): diffuse. Histological slides were digitized on a Leica Aperio AT2 digital slide scanner (Leica Biosystems, Buffalo Grove, IL, USA) at a resolution of 0.25 μm/pixel (40× objective). TIFF images were taken from the digital slides using manufacturer-provided software (ImageScope v12.4.6, Leica Biosystems). Composite images were constructed in Adobe Photoshop (release 24.1.1, San Jose, CA, USA). Image adjustments were confined to cropping and global adjustments of brightness/contrast or white balance that did not alter the interpretation of the image.

### 2.7. Isolation of Lung Cells and Flow Cytometry

Lung samples were collected in 5 mL of complete RPMI medium (RPMI + 10% FBS + antibiotics), then cut into small pieces on ice, and transferred to a 15 mL tube containing 5 mL of collagenase digestion solution (300 U/mL of collagenase type II and 1500 U DNase I in PBS). The samples were incubated at 37 °C for 1 h under constant horizontal shaking (300 rpm). Digested tissues were placed on a 75 µm sieve and washed with 10 mL of complete RPMI medium. Alternatively, lung samples were directly ground using a plunger on a nylon mesh to mitigate any effects of type II collagenase digestion on the surface markers of interest. Digested lung material was collected by centrifugation at 300× *g* for 5 min. Collected red blood cells were eliminated using RBC lysis buffer (BioLegend, San Diego, CA, USA). For surface staining, isolated cells were stained using specific antibodies—CD3 PercpCy5.5, CD62L APC, CD44 PE-Cy7, CD4 Bv510, CD8 APC-Cy7, and CD69 FITC (BioLegend)—and then washed twice with FACS staining buffer. Flow cytometry was performed on a FACS Fortessa (Becton, Dickinson and Co., Franklin Lakes, NJ, USA) using FACSDiVa software, and the data was analyzed using FlowJo software (v10; Tree Star).

### 2.8. Scanning of Mouse Lungs

**Figure 2:**
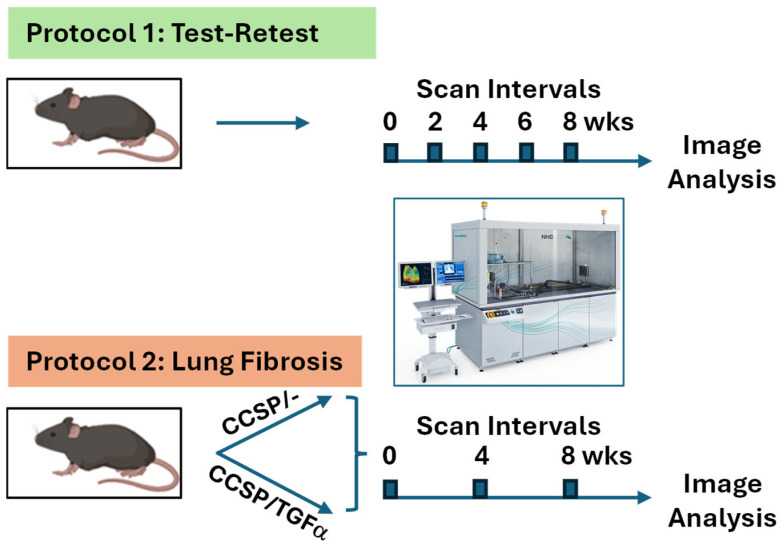
Visual representation of study design for Protocol 1 (test–retest) and Protocol 2 (lung fibrosis) longitudinal imaging studies.

The scanning protocols for the murine subjects are summarized in Figure 2 for the longitudinal test–retest study (Protocol 1) and the longitudinal lung fibrosis study (Protocol 2).

Protocol 1: Test–retest study. FVB/NJ (Jackson Laboratories, Bar Harbor, ME, USA) mice (*n* = 8) were scanned at 0, 2, 4, 6, and 8 weeks to provide a longitudinal test–retest dataset. Repeat scanning at 2-week intervals over 8 weeks was performed to assess the reproducibility of the entire scanning procedure, including mouse anesthesia and preparation, for non-diseased, age-matched mice. For each mouse, the animal body weights were also obtained over the test–retest study. The acquired scans were processed for XV quantitative measurements allowing for repeatability assessment.

Protocol 2: Lung fibrosis study. Age-matched mice consisting of two groups, CCSP/- (*n* = 9) and CCSP/TGFα (*n* = 15), were scanned at baseline (week = 0; pre-disease induction) and followed up with XV scans again at 4 and 8 weeks. The protocol was designed to evaluate the capability of the XV scanner for the longitudinal assessment of functional lung changes over time during pulmonary fibroproliferative disease progression and to determine the quantitative imaging metrics sensitive to disease progression.

### 2.9. Digital Image Processing

DICOM image files from individual mouse scans were uploaded from the scanner to the cloud for the processing of quantitative ventilation metrics by 4DMedical engineers. All data submitted to 4DMedical for processing was submitted as deidentified data; as such, the engineers did not have the key to the animal identifiers or experimental attributes. Thus, there was minimal potential for experimental bias. Then, 4DPIV was applied to quantify the heterogeneity of lung disease in mice in vivo. Regional lung ventilation was determined from 4D lung motion analysis (4DPIV) and associated with the airway structure to determine the local airflow at each airway tree branch, allowing for the detailed visualization of disease heterogeneity to provide specific regional information and quantitative metrics related to lung mechanics. Image acquisition was synchronized with mechanical ventilation, which allowed for the identification of known and corresponding time points throughout the respiratory cycle. Initial digital image post-processing involved the use of a flat-dark correction and a single-image phase retrieval routine. Pre-processed projections were then reconstructed using an algebraic tomographic reconstruction routine to obtain 3D volumes of data along the respiratory cycle. A 3-dimensional cross-correlation-based motion measurement technique was used to obtain a 3-dimensional map of lung displacement between two successive volumes in time. The cross-correlation analysis was performed with interrogation regions of 64 × 64 × 64 voxels (representing a 980 μm^3^ region) with regular spacing between the centers of adjacent interrogation regions. Analysis on reconstructed volumes resulted in displacement fields. The expansion field of lung tissue was calculated from the local gradients in the lung displacement. The expiratory time constant, which is a standard measure for studying respiratory mechanics and contains information about the mechanical properties of the lung such as the resistance and compliance, was calculated to provide quantitative metrics indicative of airway obstruction and/or reduced lung recoil as previously described [26]. The metric outputs for each animal include specific ventilation defined as the ratio of the volume of gas entering a region of the lung after an inspiration, divided by the end-expiratory volume. The specific ventilation is normalized to the mean measurement. The ventilation heterogeneity (VH), the ratio of the interquartile range to the mean specific ventilation, is used to define lung ventilation. High VH signifies substantial variability in the lungs, and low VH is typical of normal mice. VDP is calculated as the percentage of specific ventilation below 0.6 of the mean [16,27]. All the generated 4D ventilation reports and corresponding DICOM files were then uploaded back onto the cloud server for electronic transmission back to investigative team members for subsequent tabulation and evaluation (Figure 1C). The data sizes were approximately 20 GB, and the uploading time was approximately 10 min per scan file. The results for each mouse were analyzed by 4DMedical and returned as PDF and comma-separated value (.csv) files containing individual numerical values of the scan metrics for each voxel along with a complete DICOM file of both the 3D image and corresponding color-encoded quantitative ventilation map.

### 2.10. Statistical Analysis

Statistical analysis of the longitudinal FVB/NJ mouse lung test–retest imaging study’s data is provided in Supplemental Figure S1. Tabular data derived from comparing changes between various time intervals and plotted in graphical form for each measured metric (BW, VDP, MSV, TV, VH, SSVH, and LSVH) are provided. Statistical data was generated using Tukey’s multiple comparisons test.

Statistical analyses of the longitudinal CCSP/- and CCSP/TGFα lung results are provided in Supplemental Figure S2. Graphical and tabular data derived from comparing changes between various time intervals post-doxycycline induction for each measured metric (BW, VDP, MSV, TV, VH, SSVH, and LSVH) are provided. Statistical data was generated using a t-test, with *p*-values calculated for each metric comparison.

The effects of scanning protocols on imaging-derived functional lung metrics were statistically evaluated, and the results are presented in Supplemental Figure S3. Graphical and tabular data derived from comparing different scanning protocols for each measured functional lung metric (VDP, MSV, TV, VH, SSVH, and LSVH) are provided. Statistical data was generated using Tukey’s multiple comparisons test.

Protocol 1’s data, consisting of test–retest data, are reported as mean averages for each measured metric (body weight, VDP, MSV, TV, VH, SSVH, and LSVH), and the values are provided as mean (± S.E.M.) as displayed in Table 1 along with the % within-subject coefficient of variance (%wCV) defined as follows. For each metric, the longitudinal mean (M) and variance (V) were calculated over 5 timepoints to yield the V/M^2^ ratio for each mouse. The square-root of the mean of this ratio averaged over 8 mice yields the wCV, or %wCV, defined as 100% × wCV.

## 3. Results

### 3.1. Test–Retest Lung Scans

We developed methodological processes to achieve mouse intubation, the maintenance of anesthesia and ventilation, scanning, and physiological recovery, which were successfully demonstrated in 45 individual mouse-scanning procedures. The test–retest studies served to demonstrate that repetitive lung scanning studies could be accomplished with careful animal preparation that allowed for the maintenance of proper anesthesia levels and smooth ventilation cycles required by the 4DXV instrument to sync the scanner acquisition precisely with the lung ventilation cycle. Test–retest animals (*n* = 8) were scanned at five different times over a period of 8 weeks with no visual evidence of adverse effects over the study duration. Measurements of body weight and scanner-derived lung function metrics over the 8-week study are shown in Table 1 along with statistical comparisons over time (see Supplemental Data Figures S1–S3). The animal body weight significantly increased at weeks 4 to 8. The statistical evaluation of longitudinal changes for individual scanner-derived metrics revealed no change for baseline measures for ventilation defect percent (VDP), tidal volume (TV), or small-scale ventilation heterogeneity (SSVP). Mean specific ventilation (MSV) showed a significant change from baseline at week 6 (*p* < 0.05) and week 8 (*p* < 0.01). VH declined and reached significance from baseline at weeks 6 and 8 (*p* < 0.05). Large-scale ventilation heterogeneity (LSVH) declined in week 6 (*p* < 0.01) and week 8 (*p* < 0.05). The lung function changes from baseline (week 0) through week 4 revealed no significant changes over a 4-week interval, showing that the animals were stable and thus had good tolerance for the scanning procedure. Deviations from baseline outside 4 weeks may be attributed to, for example, increased body weight, age, repeat scanning, or cumulative radiation effects. Overall, these studies establish Standard Operating Procedures for the use of the 4DXV scanner for the assessment of lung function longitudinal changes; these procedures were subsequently applied to study a murine fibroproliferative lung disease model.

### 3.2. Longitudinal Assessment of Lung Fibroproliferative Disease Progression

**Figure 3:**
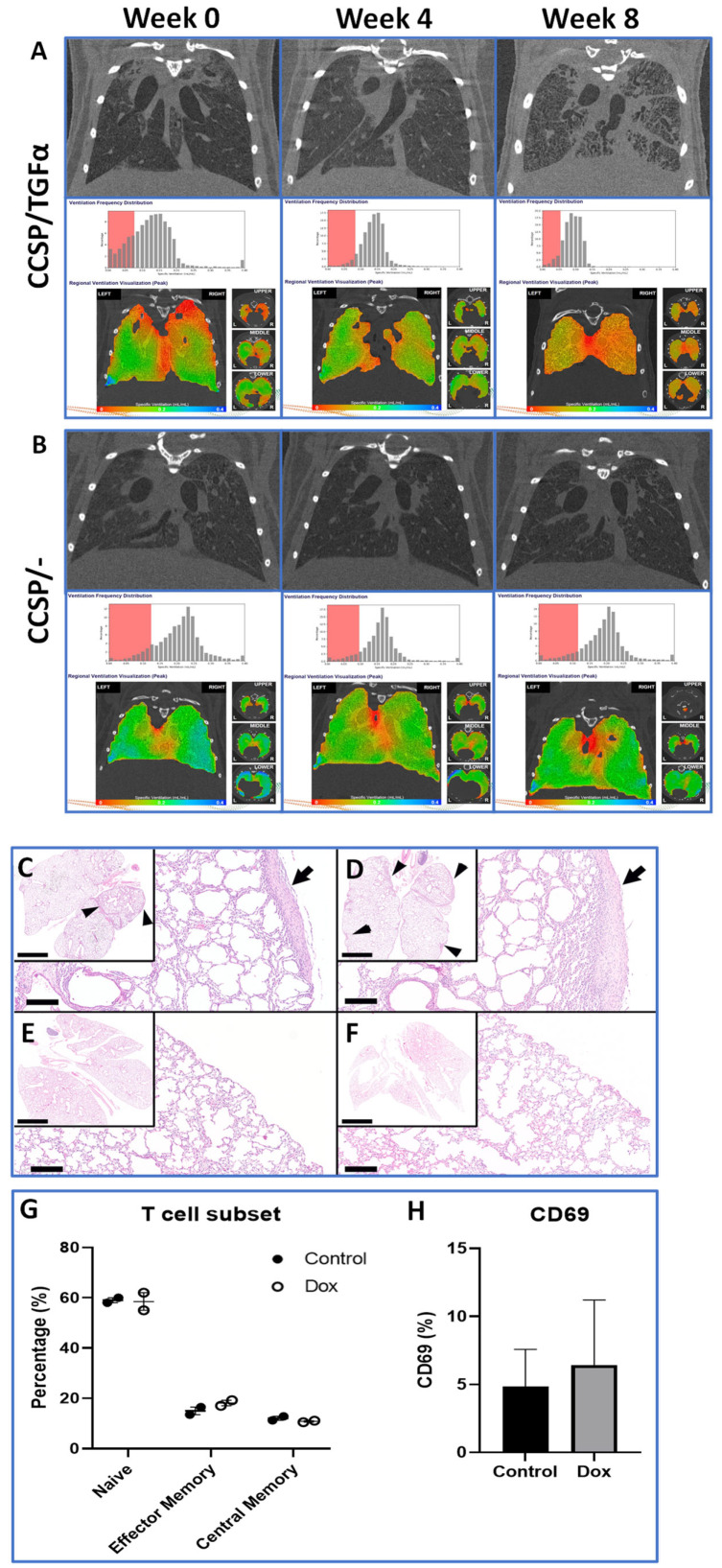
(**A**) X-ray scans of CCSP/TGFα mouse lungs at baseline (week 0), 4 weeks, and 8 weeks post-doxycycline administration. Corresponding regional ventilation heat maps and ventilation frequency distribution histograms are provided. The red box spans from 0 to 60% of the mean specific ventilation. (**B**) X-ray scans of CCSP/- mouse lungs at baseline (week 0), 4 weeks, and 8 weeks post-doxycycline administration. Corresponding regional ventilation heat maps and ventilation frequency distribution histograms are provided. The red box spans from 0 to 60% of the mean specific ventilation. Representative histological lung sections from two separate CCSP/TGFα mice (**C**,**D**) and two separate CCSP/- mice (**E**,**F**) 8 weeks post-doxycycline administration. CCSP/TGFα mice had pleural and subpleural fibrosis (arrows) multifocally in the lung lobes (arrowheads, insets). Hematoxylin and eosin staining. Bars: 3 mm and 200 μm (insets). (**G**) Lung-infiltrated immune cells collected from CCSP/- and CCSP/TGFα mice 8 weeks post-doxycycline administration with T cell subsets defined by CD44 and CD62L expression in the T cell compartment. Naïve (CD62L^+^ CD44^−^), effector memory (CD62L^−^ CD44^+^), and central memory (CD62L^+^ CD44^+^). (**H**) CD69 expression analyzed from the total T cell compartment in the isolated lung-infiltrated immune cells (*n* = 2/group). Data are means (±SEMs), and statistical significance between groups was measured using one-way ANOVA.

CCSP/TGFα mice were used to evaluate 4DXV scanning to follow functional lung changes over time compared with control CCSP/- mice. Figure 3B reveals 4DXV-derived representative images from a CCSP/- mouse along with ventilation maps scanned at time points 0, 4, and 8 weeks post-induction. Corresponding representative H&E lung tissue sections are also shown in the adjacent panels, revealing normal lung architecture, from two individual representative mice (Figure 3D,E). No significant changes in lung functional metrics were detected over time in animal controls (CCSP/-), in correspondence with the histological evaluation, which revealed no abnormal features. It is interesting to note that functional lung imaging metrics were stable over the 8-week scanning period, revealing the robust repeatability of the animal-scanning procedures.

Scans obtained at 0, 4, and 8 weeks from a representative CCSP/TGFα mouse are also displayed in Figure 3A, revealing that changes in ventilation metrics could be detected and quantified over time. Histological changes to lung parenchyma were evident (Figure 3C,D) compared with control animals (Figure 3E,F). The summary data shown in Table 2 provides quantified lung function metrics derived from the 4DXV scans for the measured time intervals (0, 4, and 8 weeks). Statistical comparisons of changes in body weight and scanner-derived lung function metrics over the 8-week study are provided (see Figure 3E). Body weight was found to decline in the CCSP/TGFα cohort as compared with the CCSP/- (control) cohort, which is typical for this lung disease model [22]. The numbers of scanned mice in the CCSP/TGFα cohort decreased from week 0 (*n* = 15) to week 8 (*n* = 4) due to attrition from progressive disease and diversion to a different protocol. Two mice were deemed too diseased to scan at 8 weeks but were included in the histological lung analysis, one mouse died after the 4-week scan for unknown reasons, one mouse died prior to the 4-week scan for unknown reasons, and seven mice were diverted to a different protocol after the 4-week scan. The numbers of mice in the CCSP/- cohort also varied over the experimental study (week 0, *n* = 9; week 4, *n* = 9; week 8, *n* = 3) as selected control mice were diverted to other protocols. Image analysis revealed that statistical changes in lung tidal volume occurred, which declined in CCSP/TGFα mice from 159.7 to 113.8 μL during fibroproliferative disease progression. This metric appears sensitive to shrinkage of the lungs and/or fibrotic restriction on lung respiratory compliance. The small-scale ventilation heterogeneity decreased (15.65% to 11.44%), which correlated with the destruction of small airways over the 8-week study period.

### 3.3. Histology and Immune Infiltrate Analysis

At the conclusion of the planned studies, lung tissue was collected from control CCSP/- (*n* = 3) and CCSP/TGFα mice (*n* = 6) for histopathological examination (Figure 3). Microscopy findings from all six samples of mouse lungs with TGFα overexpression (CCSP/TGFα) showed increased collagen deposition consistent with fibrosis in all samples. CCSP/TGFα mice were found to have pleural and subpleural fibrosis (arrows) multifocally in the lung lobes (arrowheads, insets) (Figure 3C,D). Moreover, the normal lung architecture, including the alveoli, was also disrupted, leading to thickening of the alveolar walls associated with the formation of fibrotic lesions. Most mice (*n* = 5/6) had grade 2 findings, including moderate pleural and adventitial fibrosis, while one mouse (1/6) was determined to have mild pleural and adventitial fibrosis (grade 1). Overall, the distribution of fibrosis was predominantly in pleural and peribronchial regions, with much less involvement of the interstitium (Figure 3C,D). This pattern of pleural and peribronchial fibrosis has previously been reported in mice with pulmonary overexpression of TGFα [22]. There was not a strong inflammatory response, since there were only small-to-moderate numbers of alveolar macrophages, and neutrophils were not present. Bronchus-associated lymphoid tissue (BALT) was not expanded. Tissue samples from CCSP/- mice were histologically normal with no findings (3/3) (Figure 3E,F). Subsets of T cell infiltrates were examined in a pilot flow cytometry study. No differences in percent effector T cells or percent central memory T cells were found between treatment groups, consistent with the histological examination (Figure 3G). Additionally, no change in percent CD69 levels was noted between groups (Figure 3H), which was also consistent with the lack of significant inflammatory response noted in the histological evaluation.

### 3.4. Mouse Radiation Exposure

We undertook evaluation of the delivered radiation dose in a series of scanning protocols including the standard imaging protocol, along with alternate acquisition protocols developed in this study, to lower accumulative radiation exposure. These studies focused on quantifying single-scan radiation exposure to determine the effect of X-ray dose on the accurate quantification of lung function imaging-derived metrics. Radiation dosimetry information is also important for planning and conducting repeat imaging studies, where the minimization of lung X-ray exposure is needed to limit the potential for inducing unwanted biological effects during the longitudinal scanning of murine pulmonary disease models. In these studies, sets of X-ray-sensitive films were scanned using each of the four scanning protocols summarized in Table 3. Films were affixed to the front of solid water blocks to represent the ventral surface of the mouse for each scanning protocol. Based on these studies, for the initial scanning protocol, the ventral surface of the mouse had an average dose of 439.8 cGy, and the protocol utilized 800 PPTP and a total scan time of 4:38 min. Films sandwiched between the first and second layers of solid water blocks (representing the location of the mouse lungs) had an average reduced dose of 268.6 cGy. Films affixed to the back of the solid water blocks (representing the dorsal surface of the mouse) had an average dose of 376.1 cGy. This dose is lower than those for the films on the front of the block due to the shielding effect from the plastic mouse holder. Data from the 600 PPTP protocol revealed a dose of 174.5 cGy representing lung exposure from a single scan. The results from the 450 PPTP protocol with an increased breadth rate of 220 BPM revealed an X-ray lung dose exposure of 144.8 cGy using an X-ray filter of 32 μm Mo. The lowest lung X-ray dose achieved from these studies was 81.2 cGy when using a 72 μm Mo X-ray filter (Table 3). This flexibility of the 4DXV system allows for numerous scanning protocol modifications for adapting the scanner to meet unique scanning needs.

**Figure 4:**
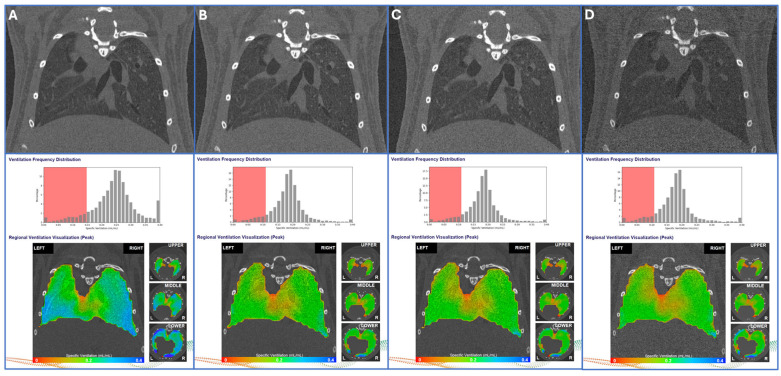
X-ray scans of CD-1 mouse lungs demonstrate the effect of acquisition parameters on ventilation metrics. Corresponding regional ventilation heat maps and ventilation frequency distribution histograms are provided. Images and frequency distributions are derived from the following imaging parameters: projections per time point (PPTP)/breadth rate (per min)/filter thickness (μm) of (**A**) 800/176/32, (**B**) 600/220/32, (**C**) 450/220/32, and (**D**) 450/220/72 as detailed in Table 3. Effect of X-ray dose on lung CT and ventilation maps. The red box spans from 0 to 60% of the mean specific ventilation.

Groups of eight mice per scanning protocol were used to evaluate each of the four protocols shown in Table 3 for quantitative functional imaging-derived metrics. For each of the scanning protocols, VDP, MSV, Tidal Volume, VH, VH Small Scale, and VH Large Scale were measured and are displayed. Importantly, for the lowest-dose exposures consisting of 450 PPTP scans, using either the 32 or 72 μm Mo X-ray filters, each scan set had nearly identical quantitative functional lung metrics, which were also consistent with the 600 PPTP scanning protocol. Only the higher-dose protocol (800 PPTP) had elevated values across the functional lung metrics. Visualization of the quantitative imaging maps and corresponding Ventilation Frequency Distribution histograms appeared similar across each of Table 3’s image acquisition protocols as shown in Figure 4A–D. Statistical comparisons of the three newly derived scanning protocols compared to the PPTP 800 protocol revealed that, for each protocol, the measurements of SSVH, TV, and MSV were significantly lower but consistent between the lower-dose protocols.

## 4. Discussion

Acquiring preclinical longitudinal lung imaging data across more than one time interval for an individual animal using the 4XVD technology has not previously been reported. Currently, mice are tracheotomized to allow for direct connection to the scanner ventilator; therefore, reported studies consist of end-stage, non-survival scans. Here, we describe the development and demonstration of mouse-scanning protocols specifically allowing for routine animal scanning and post-scan recovery to achieve longitudinal scanning (Figure 1). The longitudinal scanning of individual animals provided a key opportunity to undertake the test–retest scanning of a cohort of animals over two months to evaluate the stability and reproducibility of the dynamic lung function measurements. This study also revealed the robustness of the anesthesia protocol to provide for longitudinal mouse scanning. Repeat scanning capability is especially important, as imaging has significant potential to enable noninvasive and detailed lung assessment to characterize the extent of the disease trajectory and its impact on lung function in an individual animal over the course of a study.

As shown in Table 1, the repeatability of functional lung metrics from FVB/NJ mice imaged every two weeks over two months was evaluated. The statistics of repeatability for a given metric were determined by the within-subject coefficient of variance (wCV), which is calculated from the longitudinal variance of each mouse relative to its squared-mean and then averaged over all mice [28]. In this study, test–retest analysis was used to assess the technical precision in measuring the lung metrics, assuming that the true biological change between the test and retest may be considered small. An empirically determined wCV value provided the reference value that must be well exceeded for a true biological change detected via the metric to be biologically significant for an individual animal. Overall, we found that the XV scanner performed consistently, demonstrating good repeatability over the two-month repeat scan experimental duration.

Pulmonary fibrosis is a significant health problem contributing to morbidity and mortality in patient populations [29,30,31,32]. In this study, a transgenic mouse model of pulmonary fibroproliferative disease, leading to progressive fibrosis independent of inflammation or inflammatory cell infiltrates, was scanned over time to characterize the state of disease-induced changes in lung function. Figure 3 reveals that changes in ventilation frequency distribution were detectable at 4 weeks post-induction, with further progressive changes at 8 weeks for CCSP/TGFα mice compared to CCSP/- mice. XV-derived quantitative metrics revealed decreases in VDP, tidal volume, and small-scale ventilation heterogeneity for the CCSP/TGFα cohort. VDP is a measure of regional airway obstruction and air trapping commonly used, for example, to assess asthma. For the CCSP/TGFα model, this metric decreased due to tissue destruction. Moreover, the observed decreases in small-scale ventilation heterogeneity (SSVH) and tidal volume for CCSP/TGFα animals are associated with lung fibrosis and the loss of organized, functional lung parenchyma. The progressive increase in large-scale ventilation heterogeneity observed in the CCSP/TGFα cohort suggests spatially varying regions of fibrosis within the lung parenchyma. While the CCSP/TGFα model used herein provided an opportunity to initiate validation of the image acquisition protocols developed herein for repeat measurements, a follow-up cohort of animals with more frequent scanning intervals would likely be needed to further elucidate spatiotemporal changes in lung function due to fibroproliferative disease due to untimely deaths attributed to late-stage disease progression.

The results obtained using XV technology were consistent with a prior report which used MRI which revealed increased pleural lung thickness along with decreased lung tidal volume in CCSP/TGFα mice [33]. While validation studies comparing XV metrics to traditional lung function tests in animals are underway, there are several publications for human subjects reporting XV validation against PFTs [34].

Our development of optimized image acquisition protocols using the 4DMedical XV lung scanner enabled repeat lung scanning longitudinally. The evaluation of multiple scanning protocols revealed that a reduction in the delivered X-ray dose for individual scans could be achieved while maintaining the image quality necessary for undertaking functional analysis of the acquired images. While it was noted that lower-dose protocols could affect the numerical values of specific functional lung metrics, using the same protocol over time should provide a key opportunity to minimize the delivered X-ray dose. The evaluation of the impact of scanning parameters allowed for further reducing the radiation dose for a single scan to decrease the net cumulative X-ray dose received from a multi-scan study. However, statistical analysis revealed that different protocols may affect specific measure ventilation metrics; thus, consistent use of metrics within an experiment should be maintained. The scanner is equipped with a filter wheel consisting of different thicknesses of Mo between the X-ray source and the animal holder. This feature allows for direct adjustment of the dose delivered by the source without having to change the scan time, breadth rate, or PPTP. X-ray dosimetry studies revealed that scans with sufficient resolution and sensitivity for functional lung analysis ranged from 268.6 cGy to 81.2 cGy using our scan parameters. Thus, at the lowest dose evaluated, mice receiving a total of three scans would have an accumulated total radiation dose of 243.6 cGy. Additional X-ray dose reductions are likely achievable if needed for more sensitive animal models. Additional consideration should be given to literature reports which suggest that X-ray dosages produce biological effects that are dependent on mouse strain [35,36,37,38,39,40,41,42,43], age [44], health status [45], time of day [46], and the time interval between doses [47]. As radiation can result in DNA damage, oxidative stress, and pro-inflammatory responses which can alter cellular and mitochondrial redox balance, form oxidized nucleotides, and impair DNA repair [48], careful evaluation of overall accumulated X-ray doses should be considered.

## 5. Conclusions

We demonstrated that longitudinal scanning of mice using the XV scanner can be achieved through careful consideration of the anesthesia preparation and recovery, in addition to the scanner acquisition settings. Limitations which may affect the generalization of our findings include variations amongst mouse strains and age, the extent of disease status, the limited sample size, and other confounding factors including the potential for image-processing variations. The versatility and flexibility in adjusting the 4DXV acquisition parameters provide for significant refinement and customization of the scanner for the optimization of the image resolution, X-ray dose exposure, breadth rate, and number of projections collected. The software settings allow for numerous adaptations to be made to suit individual specific requirements of experimental mouse models and study protocols. Moreover, the 4DXV technology was validated in first-in-human studies which revealed the visualization of local variations in dysfunctional lung ventilation that were not detectable using standard pulmonary functional tests (PFTs) or CT measurements [49]. More recently, patient studies further demonstrated XV’s safety and value for examining lung function [50,51]. Overall, emerging results continue to suggest that 4DXV has potential for contributing significant and important new biomarker metrics to clinical composite classification scores [52]. The ability to apply XV technology from mouse to human lung exams provides exciting and important new opportunities for conducting co-clinical trials to evaluate this technology across the continuum of lung disorders and array of therapeutic interventions.

## Figures and Tables

**Table 1:** Test–retest data averages and wCV.

Metric	Week 0	Week 2	Week 4	Week 6	Week 8	wCV (%)
Body Weight (g)	20.19 (0.34)	21.35 (0.32)	22.36 (0.28)	23.69 (0.37)	23.99 (0.29)	-
VDP (%)	10.84 (0.82)	10.37 (0.45)	10.22 (0.38)	8.89 (0.68)	8.62 (0.32)	17.59
MSV (mL/mL)	219.5 (4.3)	203.6 (7.0)	202.1 (4.7)	192.9 (7.2)	190.8 (3.2)	8.00
Tidal Volume (mL)	137.9 (2.0)	143.5 (4.5)	144.9 (1.9)	144.4 (3.2)	144.0 (1.7)	5.33
VH (%)	33.38 (1.64)	32.49 (1.09)	31.65 (0.55)	28.33 (1.20)	28.40 (0.76)	11.91
VH–Small Scale (%)	16.34 (0.86)	15.96 (0.66)	16.13 (0.32)	14.84 (0.75)	14.22 (0.43)	11.40
VH–Large Scale (%)	23.92 (1.20)	22.71 (1.31)	21.42 (0.46)	18.26 (0.96)	19.81 (0.66)	15.98

Mean (±S.E.M.). VDP—ventilation defect percent; MSV—mean specific ventilation; VH—ventilation heterogeneity.

**Table 2:** Longitudinal bodyweight and imaging metrics.

Metric	CCSP/	Week 0	Week 4	Week 8
Body Weight (g)	-	26.64 (0.94)	28.93 (1.52)	30.33 (2.60)
	TGFα	24.87 (0.66)	26.12 (1.01)	22.25 (1.14)
Ventilation Defect Percentage (VDP %)	-	11.02 (0.75)	10.96 (0.70)	10.01 (0.44)
	TGFα	12.32 (0.91)	11.58 (1.81)	8.99 (1.25)
Mean Specific Ventilation (MSV, mL/mL)	-	179.7 (5.0)	179.7 (5.7)	175.0 (8.5)
	TGFα	125.0 (7.6)	132.1 (6.4)	134.6 (16.1)
Tidal Volume (mL)	-	155.4 (4.7)	167.6 (6.6)	187.7 (2.2)
	TGFα	159.7 (2.7)	155.8 (5.8)	113.8 (21.1)
Ventilation Heterogeneity (%)	-	34.64 (2.53)	33.91 (1.98)	31.65 (1.21)
	TGFα	39.61 (2.29)	40.76 (5.12)	39.72 (3.06)
Small-Scale Ventilation Heterogeneity (%)	-	14.96 (0.69)	14.96 (077)	14.50 (1.07)
	TGFα	15.65 (0.59)	13.93 (1.00)	11.44 (0.50)
Large-Scale Ventilation Heterogeneity (%)	-	24.20 (1.98)	23.23 (1.58)	19.76 (0.70)
	TGFα	28.72 (2.15)	29.66 (4.06)	32.37 (3.61)

Mean (±S.E.M.).

**Table 3:** Effects of scan parameters on ventilation metrics and lung irradiation dose.

Scan Rate (PPTP)	Breadth Rate (per min)	X-Ray Filter (mm Mo)	Scan Time (min)	X-Ray Dose (cGY)	VDP (%)	MSV (mL/mL)	Tidal Volume (mL)	VH (%)	VH Small-Scale (%)	VH Large-Scale (%)
800	176	32	4:38	268.6	10.08 (0.53)	238.4 (6.9)	159.8 (4.9)	31.05 (1.00)	14.80 (0.39)	21.11 (0.49)
600	220	32	2:47	174.5	8.55 (0.45)	177.3 (5.4)	123.8 (3.6)	27.18 (1.04)	12.34 (0.43)	18.84 (0.51)
450	220	32	2:05	144.8	8.81 (0.48)	176.5 (5.4)	122.8 (3.4)	27.49 (1.13)	12.46 (0.40)	19.24 (0.63)
450	220	72	2:05	81.2	9.26 (0.47)	175.9 (4.9)	121.4 (3.3)	27.88 (0.92)	12.85 (0.48)	19.07 (0.56)

Definition of abbreviations: PPTP = projections per time point; VDP = ventilation defect percent; MSV = mean specific ventilation; VH = ventilation heterogeneity. *n*= 8. Mean (±S.E.M.).

## Data Availability

The data generated or analyzed during this study are included in this published article (and its Supplementary Information files), and additional datasets generated during the current study are available from the corresponding author on reasonable request.

## Supplementary Materials

The following supporting information can be downloaded at: https://www.mdpi.com/article/10.3390/tomography11100112/s1. Figure S1: Statistical analysis of the longitudinal FVB/NJ mouse lung test-retest imaging study; Figure S2: Statistical analysis of the longitudinal CCSP/- and CCSP/TGFa lung results; Figure S3: Effects of scanning protocols on imaging-derived functional lung metrics.

## Glossary

DICOM = digital imaging and communications in medicine; 4DPIV = volumetric particle image velocimetry; 4DXV = four-dimensional X-ray velocimetry; FEF = forced expired flow; FEV = forced expired volume; FVC = forced vital capacity; LSVP = large-scale ventilation percent; MSV = mean specific ventilation; PPTP = projections per time point; SSVP = small-scale ventilation heterogeneity; TV = tidal volume; VDP = ventilation defect percent; VH = ventilation heterogeneity; percent within-subject coefficient of variance (%wCV); XV = X-ray velocimetry.

## References

[1] McGovern T.K., Robichaud A., Fereydoonzad L., Schuessler T.F., Martin J.G. (2013). Evaluation of respiratory system mechanics in mice using the forced oscillation technique. J. Vis. Exp..

[2] De Guia R.M., Zatecka V., Rozman J., Prochazka J., Sedlacek R. (2022). Full Assessment of Lung Mechanics Using Computer-Controlled, Forced Oscillation Technique. Curr. Protoc..

[3] Shofer S., Badea C., Auerbach S., Schwartz D.A., Johnson G.A. (2007). A micro-computed tomography-based method for the measurement of pulmonary compliance in healthy and bleomycin-exposed mice. Exp. Lung Res..

[4] Redente E.F., Kopf K.W., Bahadur A.N., Robichaud A., Lundblad L.K., McDonald L.T. (2023). Application-specific approaches to MicroCT for evaluation of mouse models of pulmonary disease. PLoS ONE.

[5] Parker J.C. (2011). Acute lung injury and pulmonary vascular permeability: Use of transgenic models. Compr. Physiol..

[6] Tielemans B., Dekoster K., Verleden S.E., Sawall S., Leszczynski B., Laperre K., Vanstapel A., Verschakelen J., Kachelriess M., Verbeken E. (2020). From Mouse to Man and Back: Closing the Correlation Gap between Imaging and Histopathology for Lung Diseases. Diagnostics.

[7] Schuster D.P., Kovacs A., Garbow J., Piwnica-Worms D. (2004). Recent advances in imaging the lungs of intact small animals. Am. J. Respir. Cell Mol. Biol..

[8] Galban C.J., Han M.K., Boes J.L., Chughtai K.A., Meyer C.R., Johnson T.D., Galban S., Rehemtulla A., Kazerooni E.A., Martinez F.J. (2012). Computed tomography-based biomarker provides unique signature for diagnosis of COPD phenotypes and disease progression. Nat. Med..

[9] Kauczor H., Surkau R., Roberts T. (1998). MRI using hyperpolarized noble gases. Eur. Radiol..

[10] Dugas J.P., Garbow J.R., Kobayashi D.K., Conradi M.S. (2004). Hyperpolarized (3)He MRI of mouse lung. Magn. Reson. Med..

[11] Niedbalski P.J., Cochran A.S., Freeman M.S., Guo J., Fugate E.M., Davis C.B., Dahlke J., Quirk J.D., Varisco B.M., Woods J.C. (2021). Validating in vivo hyperpolarized (129) Xe diffusion MRI and diffusion morphometry in the mouse lung. Magn. Reson. Med..

[12] Wakayama T., Ueyama T., Imai F., Kimura A., Fujiwara H. (2022). Quantitative assessment of regional lung ventilation in emphysematous mice using hyperpolarized (129)Xe MRI with a continuous flow hyperpolarizing system. Magn. Reson. Imaging.

[13] Karmali D., Sowho M., Bose S., Pearce J., Tejwani V., Diamant Z., Yarlagadda K., Ponce E., Eikelis N., Otvos T. (2023). Functional imaging for assessing regional lung ventilation in preclinical and clinical research. Front. Med..

[14] Murrie R.P., Paganin D.M., Fouras A., Morgan K.S. (2016). Phase contrast x-ray velocimetry of small animal lungs: Optimising imaging rates. Biomed. Opt. Express.

[15] Werdiger F., Donnelley M., Dubsky S., Murrie R.P., Carnibella R.P., Samarage C.R., How Y.Y., Zosky G.R., Fouras A., Parsons D.W. (2020). Quantification of muco-obstructive lung disease variability in mice via laboratory X-ray velocimetry. Sci. Rep..

[16] Asosingh K., Frimel M., Zlojutro V., Grant D., Stephens O., Wenger D., Fouras A., DiFilippo F., Erzurum S. (2022). Preclinical Four-Dimensional Functional Lung Imaging and Quantification of Regional Airflow: A New Standard in Lung Function Evaluation in Murine Models. Am. J. Respir. Cell Mol. Biol..

[17] Reyne N., Smith R., Cmielewski P., Eikelis N., Nilsen K., Louise J., Duerr J., Mall M.A., Lawrence M., Parsons D. (2025). Functional Lung Imaging Identifies Peripheral Ventilation Changes in β-ENaC Mice. Respirology.

[18] Reyne N., Smith R., Cmielewski P., Eikelis N., Lawrence M., Louise J., Pirakalathanan P., Parsons D., Donnelley M. (2024). Assessment of respiratory mechanics and X-ray velocimetry functional imaging in two cystic fibrosis rat models. Sci. Rep..

[19] Constantinou C., Attix F.H., Paliwal B.R. (1982). A solid water phantom material for radiotherapy x-ray and gamma-ray beam calibrations. Med. Phys..

[20] Levin D.S., Friedman P.S., Ferretti C., Ristow N., Tecchio M., Litzenberg D.W., Bashkirov V., Schulte R. (2024). A Prototype Scintillator Real-Time Beam Monitor for Ultra-high Dose Rate Radiotherapy. Med. Phys..

[21] Tichelaar J.W., Lu W., Whitsett J.A. (2000). Conditional expression of fibroblast growth factor-7 in the developing and mature lung. J. Biol. Chem..

[22] Hardie W.D., Le Cras T.D., Jiang K., Tichelaar J.W., Azhar M., Korfhagen T.R. (2004). Conditional expression of transforming growth factor-alpha in adult mouse lung causes pulmonary fibrosis. Am. J. Physiol. Lung Cell. Mol. Physiol..

[23] Gage G.J., Kipke D.R., Shain W. (2012). Whole animal perfusion fixation for rodents. J. Vis. Exp..

[24] Thomas S.M., Bednarek J., Janssen W.J., Hume P.S. (2021). Air-Inflation of Murine Lungs with Vascular Perfusion-Fixation. J. Vis. Exp..

[25] Renne R., Brix A., Harkema J., Herbert R., Kittel B., Lewis D., March T., Nagano K., Pino M., Rittinghausen S. (2009). Proliferative and nonproliferative lesions of the rat and mouse respiratory tract. Toxicol. Pathol..

[26] Stahr C.S., Samarage C.R., Donnelley M., Farrow N., Morgan K.S., Zosky G., Boucher R.C., Siu K.K., Mall M.A., Parsons D.W. (2016). Quantification of heterogeneity in lung disease with image-based pulmonary function testing. Sci. Rep..

[27] Diamond V.M., Bell L.C., Bone J.N., Driehuys B., Menchaca M., Santyr G., Svenningsen S., Thomen R.P., Marshall H., Smith L.J. (2025). A Systematic Review of the Variability of Ventilation Defect Percent Generated From Hyperpolarized Noble Gas Pulmonary Magnetic Resonance Imaging. J. Magn. Reason. Imaging.

[28] Raunig D.L., McShane L.M., Pennello G., Gatsonis C., Carson P.L., Voyvodic J.T., Wahl R.L., Kurland B.F., Schwarz A.J., Gonen M. (2015). Quantitative imaging biomarkers: A review of statistical methods for technical performance assessment. Stat. Methods Med. Res..

[29] Martinez F.J., Collard H.R., Pardo A., Raghu G., Richeldi L., Selman M., Swigris J.J., Taniguchi H., Wells A.U. (2017). Idiopathic pulmonary fibrosis. Nat. Rev. Dis. Primers.

[30] Muri J., Durcova B., Slivka R., Vrbenska A., Makovicka M., Makovicky P., Skarda J., Delongova P., Kamarad V., Vecanova J. (2024). Idiopathic Pulmonary Fibrosis: Review of Current Knowledge. Physiol. Res..

[31] Wijsenbeek M., Cottin V. (2020). Spectrum of Fibrotic Lung Diseases. N. Engl. J. Med..

[32] Wu X., Kim G.H., Salisbury M.L., Barber D., Bartholmai B.J., Brown K.K., Conoscenti C.S., De Backer J., Flaherty K.R., Gruden J.F. (2019). Computed Tomographic Biomarkers in Idiopathic Pulmonary Fibrosis. The Future of Quantitative Analysis. Am. J. Respir. Crit. Care Med..

[33] Guo J., Hardie W.D., Cleveland Z.I., Davidson C., Xu X., Madala S.K., Woods J.C. (2019). Longitudinal free-breathing MRI measurement of murine lung physiology in a progressive model of lung fibrosis. J. Appl. Physiol..

[34] Karmali D., Afanador-Castiblanco S., Otvos T., Aguilar G., Hossen S., Eikelis N., Nilsen K., Punjabi N.M., Siddharthan T., Kirkness J.P. (2025). Regional lung volume changes with noninvasive positive pressure ventilation in healthy adults. J. Appl. Physiol..

[35] Kallman R.F., Kohn H.I. (1956). The influence of strain on acute x-ray lethality in the mouse. I. LD50 and death rate studies. Radiat. Res..

[36] Grahn D., Hamilton K.F. (1957). Genetic Variation in the Acute Lethal Response of Four Inbred Mouse Strains to Whole Body X-Irradiation. Genetics.

[37] Grahn D. (1958). Acute Radiation Response of Mice from a Cross between Radiosensitive and Radioresistant Strains. Genetics.

[38] Mori N., Okumoto M., Morimoto J., Imai S., Matsuyama T., Takamori Y., Yagasaki O. (1992). Genetic analysis of susceptibility to radiation-induced apoptosis of thymocytes in mice. Int. J. Radiat. Biol..

[39] Iwakawa M., Noda S., Ohta T., Ohira C., Lee R., Goto M., Wakabayashi M., Matsui Y., Harada Y., Imai T. (2003). Different radiation susceptibility among five strains of mice detected by a skin reaction. J. Radiat. Res..

[40] Ohta T., Iwakawa M., Oohira C., Noda S., Minfu Y., Goto M., Tanaka H., Harada Y., Imai T. (2004). Fractionated irradiation augments inter-strain variation of skin reactions among three strains of mice. J. Radiat. Res..

[41] Biedermann K.A., Sun J.R., Giaccia A.J., Tosto L.M., Brown J.M. (1991). scid mutation in mice confers hypersensitivity to ionizing radiation and a deficiency in DNA double-strand break repair. Proc. Natl. Acad. Sci. USA.

[42] Jackson I.L., Vujaskovic Z., Down J.D. (2010). Revisiting strain-related differences in radiation sensitivity of the mouse lung: Recognizing and avoiding the confounding effects of pleural effusions. Radiat. Res..

[43] Boria A.J., Perez-Torres C.J. (2020). Impact of mouse strain and sex when modeling radiation necrosis. Radiat. Oncol..

[44] Spalding J.F., Trujillo T.T. (1962). Radiosensitivity of mice as a function of age. Radiat. Res..

[45] Nakajima T., Ninomiya Y., Unno K., Morioka T., Nishimura M., Kakinuma S. (2022). Impacts of psychological stress on high dose-rate radiation acute effects in a mouse experimental model. J. Radiat. Res..

[46] Pizzarello D.J., Witcofski R.L. (1970). A possible link between diurnal variations in radiation sensitivity and cell division in bone marrow of male mice. Radiology.

[47] Cui Y.Z., Hisha H., Yang G.X., Fan T.X., Jin T., Li Q., Lian Z., Ikehara S. (2002). Optimal protocol for total body irradiation for allogeneic bone marrow transplantation in mice. Bone Marrow Transplant..

[48] Shin E., Lee S., Kang H., Kim J., Kim K., Youn H., Jin Y.W., Seo S., Youn B. (2020). Organ-Specific Effects of Low Dose Radiation Exposure: A Comprehensive Review. Front. Genet..

[49] Bruorton M., Donnelley M., Goddard T., O’Connor A., Parsons D., Phillips J., Carson-Chahhoud K., Tai A. (2024). Pilot study of paediatric regional lung function assessment via X-ray velocimetry (XV) imaging in children with normal lungs and in children with cystic fibrosis. BMJ Open.

[50] Kirkness J.P., Dusting J., Eikelis N., Pirakalathanan P., DeMarco J., Shiao S.L., Fouras A. (2023). Association of x-ray velocimetry (XV) ventilation analysis compared to spirometry. Front. Med. Technol..

[51] Siddharthan T., Grealis K., Kirkness J.P., Ötvös T., Stefanovski D., Tombleson A., Dalzell M., Gonzalez E., Nakrani K.B., Wenger D. (2023). Quantifying ventilation by X-ray velocimetry in healthy adults. Respir. Res..

[52] Richmond B.W., Lester M.G., Lui V., Dusting J., Raju S., Snell G.I., Blackburn J.B., Douglas K., Miller R.F., Siddharthan T. (2025). X-ray velocimetry provides temporally and spatially-resolved biomarkers of lung ventilation in small airways disease. Respir. Res..

