# Supplementary material for: Murine Functional Lung Imaging Using X-Ray Velocimetry for Longitudinal Noninvasive Quantitative Spatial Assessment of Pulmonary Airflow
Source: Tomography. 2025 Oct 2;11(10):112. doi: 10.3390/tomography11100112 (PMC12567626; doi:10.3390/tomography11100112)
Supplement: Supplementary file 1 [file tomography-11-00112-s001.zip › tomography-3866203-supplementary.pdf]

Murine Functional Lung Imaging for Longitudinal Noninvasive Quantitative Spatial Assessment of Pulmonary Airflow

Kevin A. Heist[1], Christopher A. Bonham[1], Youngsoon Jang[1], Ingrid L. Bergin[2], Amanda Welton[1], David Karnak[3], Charles A. Hatt[4], Matthew Cooper[4], Wilson Teng[4], William D. Hardie[5], Thomas L. Chenevert[1] and Brian D. Ross[1,6*]

Online Data Supplement

Figure S1: Statistical analysis of the longitudinal FVB/NJ mouse lung test-retest imaging study. Tabular data derived from comparing changes between various time intervals and plotted in graphical form for each measured metric (BW, VDP, MSV, TV, VH, SSVH, and LSVH).

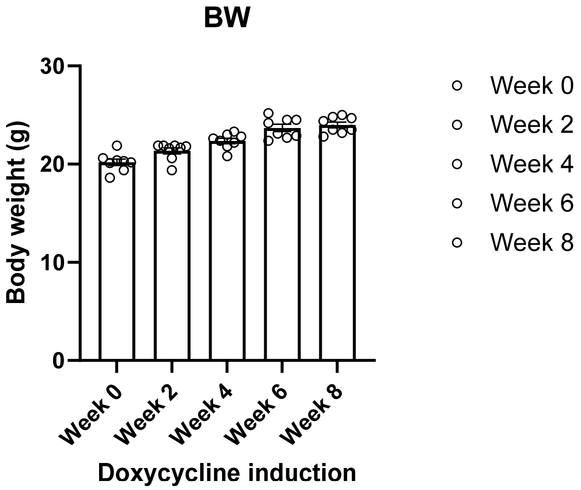

Tukey's multiple comparisons test	Mean Diff.	95.00% CI of diff.	Below threshold?	Summary	Adjusted P Value
Week 0 vs. Week 2	-1.163	-2.463 to 0.1375	No	ns	0.0981
Week 0 vs. Week 4	-2.175	-3.475 to -0.8750	Yes	***	0.0003
Week 0 vs. Week 6	-3.500	-4.800 to -2.200	Yes	****	<0.0001
Week 0 vs. Week 8	-3.800	-5.100 to -2.500	Yes	****	<0.0001
Week 2 vs. Week 4	-1.013	-2.313 to 0.2875	No	ns	0.1894
Week 2 vs. Week 6	-2.338	-3.638 to -1.037	Yes	****	<0.0001
Week 2 vs. Week 8	-2.638	-3.938 to -1.337	Yes	****	<0.0001
Week 4 vs. Week 6	-1.325	-2.625 to -0.02496	Yes	*	0.0440
Week 4 vs. Week 8	-1.625	-2.925 to -0.3250	Yes	**	0.0083
Week 6 vs. Week 8	-0.3000	-1.600 to 1.000	No	ns	0.9629
Test details	Mean 1	Mean 2	Mean Diff.	SE of diff.	n1
Week 0 vs. Week 2	20.19	21.35	-1.163	0.4522	8
Week 0 vs. Week 4	20.19	22.36	-2.175	0.4522	8
Week 0 vs. Week 6	20.19	23.69	-3.500	0.4522	8
Week 0 vs. Week 8	20.19	23.99	-3.800	0.4522	8
Week 2 vs. Week 4	21.35	22.36	-1.013	0.4522	8
Week 2 vs. Week 6	21.35	23.69	-2.338	0.4522	8
Week 2 vs. Week 8	21.35	23.99	-2.638	0.4522	8
Week 4 vs. Week 6	22.36	23.69	-1.325	0.4522	8
Week 4 vs. Week 8	22.36	23.99	-1.625	0.4522	8
Week 6 vs. Week 8	23.69	23.99	-0.3000	0.4522	8

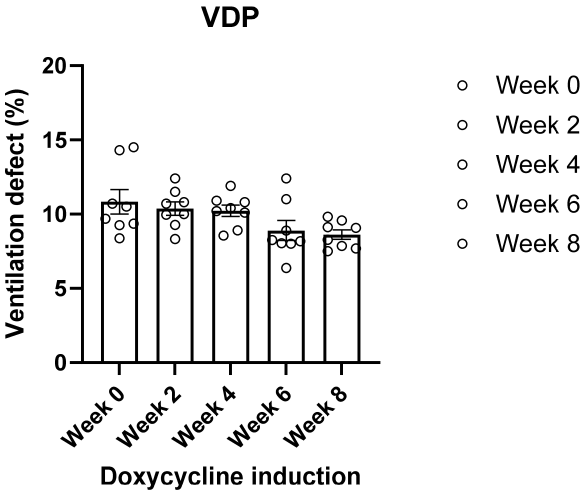

Tukey's multiple comparisons test	Mean Diff.	95.00% CI of diff.	Below threshold?	Summary	Adjusted P Value
Week 0 vs. Week 2	0.4663	-1.817 to 2.750	No	ns	0.9761
Week 0 vs. Week 4	0.6138	-1.670 to 2.897	No	ns	0.9367
Week 0 vs. Week 6	1.943	-0.3408 to 4.226	No	ns	0.1270
Week 0 vs. Week 8	2.218	-0.06576 to 4.501	No	ns	0.0604
Week 2 vs. Week 4	0.1475	-2.136 to 2.431	No	ns	0.9997
Week 2 vs. Week 6	1.476	-0.8070 to 3.760	No	ns	0.3577
Week 2 vs. Week 8	1.751	-0.5320 to 4.035	No	ns	0.2016
Week 4 vs. Week 6	1.329	-0.9545 to 3.612	No	ns	0.4630
Week 4 vs. Week 8	1.604	-0.6795 to 3.887	No	ns	0.2781
Week 6 vs. Week 8	0.2750	-2.008 to 2.558	No	ns	0.9968
Test details	Mean 1	Mean 2	Mean Diff.	SE of diff.	n1
Week 0 vs. Week 2	10.84	10.37	0.4663	0.7942	8
Week 0 vs. Week 4	10.84	10.22	0.6138	0.7942	8
Week 0 vs. Week 6	10.84	8.893	1.943	0.7942	8
Week 0 vs. Week 8	10.84	8.618	2.218	0.7942	8
Week 2 vs. Week 4	10.37	10.22	0.1475	0.7942	8
Week 2 vs. Week 6	10.37	8.893	1.476	0.7942	8
Week 2 vs. Week 8	10.37	8.618	1.751	0.7942	8
Week 4 vs. Week 6	10.22	8.893	1.329	0.7942	8
Week 4 vs. Week 8	10.22	8.618	1.604	0.7942	8
Week 6 vs. Week 8	8.893	8.618	0.2750	0.7942	8

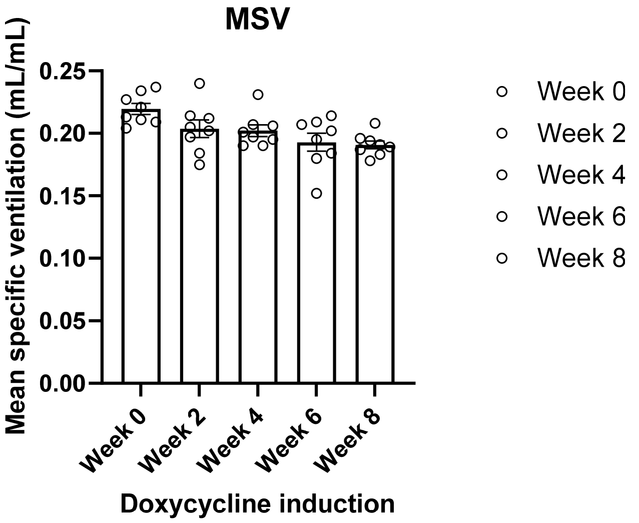

Tukey's multiple comparisons tes	Mean Diff.	95.00% CI of diff.	Below threshold?	Summary	Adjusted P Value
Week 0 vs. Week 2	0.01588	-0.006558 to 0.03831	No	ns	0.2712
Week 0 vs. Week 4	0.01738	-0.005058 to 0.03981	No	ns	0.1938
Week 0 vs. Week 6	0.02663	0.004192 to 0.04906	Yes	*	0.0133
Week 0 vs. Week 8	0.02875	0.006317 to 0.05118	Yes	**	0.0065
Week 2 vs. Week 4	0.001500	-0.02093 to 0.02393	No	ns	0.9997
Week 2 vs. Week 6	0.01075	-0.01168 to 0.03318	No	ns	0.6455
Week 2 vs. Week 8	0.01288	-0.009558 to 0.03531	No	ns	0.4768
Week 4 vs. Week 6	0.009250	-0.01318 to 0.03168	No	ns	0.7595
Week 4 vs. Week 8	0.01138	-0.01106 to 0.03381	No	ns	0.5957
Week 6 vs. Week 8	0.002125	-0.02031 to 0.02456	No	ns	0.9987
Test details	Mean 1	Mean 2	Mean Diff.	SE of diff.	n1
Week 0 vs. Week 2	0.2195	0.2036	0.01588	0.007803	8
Week 0 vs. Week 4	0.2195	0.2021	0.01738	0.007803	8
Week 0 vs. Week 6	0.2195	0.1929	0.02663	0.007803	8
Week 0 vs. Week 8	0.2195	0.1908	0.02875	0.007803	8
Week 2 vs. Week 4	0.2036	0.2021	0.001500	0.007803	8
Week 2 vs. Week 6	0.2036	0.1929	0.01075	0.007803	8
Week 2 vs. Week 8	0.2036	0.1908	0.01288	0.007803	8
Week 4 vs. Week 6	0.2021	0.1929	0.009250	0.007803	8
Week 4 vs. Week 8	0.2021	0.1908	0.01138	0.007803	8
Week 6 vs. Week 8	0.1929	0.1908	0.002125	0.007803	8

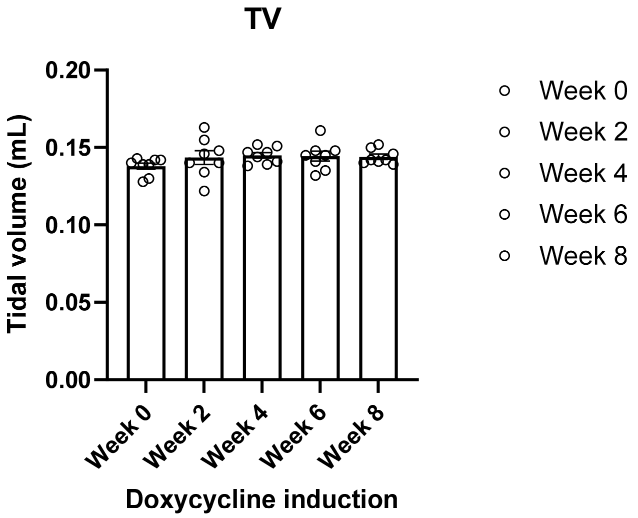

Tukey's multiple comparisons test	Mean Diff.	95.00% CI of diff.	Below threshold?	Summary	Adjusted P Value
Week 0 vs. Week 2	-0.005625	-0.01717 to 0.005915	No	ns	0.6309
Week 0 vs. Week 4	-0.007000	-0.01854 to 0.004540	No	ns	0.4214
Week 0 vs. Week 6	-0.006500	-0.01804 to 0.005040	No	ns	0.4955
Week 0 vs. Week 8	-0.006125	-0.01767 to 0.005415	No	ns	0.5532
Week 2 vs. Week 4	-0.001375	-0.01292 to 0.01017	No	ns	0.9969
Week 2 vs. Week 6	-0.0008750	-0.01242 to 0.01067	No	ns	0.9995
Week 2 vs. Week 8	-0.0005000	-0.01204 to 0.01104	No	ns	>0.9999
Week 4 vs. Week 6	0.0005000	-0.01104 to 0.01204	No	ns	>0.9999
Week 4 vs. Week 8	0.0008750	-0.01067 to 0.01242	No	ns	0.9995
Week 6 vs. Week 8	0.0003750	-0.01117 to 0.01192	No	ns	>0.9999
Test details	Mean 1	Mean 2	Mean Diff.	SE of diff.	n1
Week 0 vs. Week 2	0.1379	0.1435	-0.005625	0.004014	8
Week 0 vs. Week 4	0.1379	0.1449	-0.007000	0.004014	8
Week 0 vs. Week 6	0.1379	0.1444	-0.006500	0.004014	8
Week 0 vs. Week 8	0.1379	0.1440	-0.006125	0.004014	8
Week 2 vs. Week 4	0.1435	0.1449	-0.001375	0.004014	8
Week 2 vs. Week 6	0.1435	0.1444	-0.0008750	0.004014	8
Week 2 vs. Week 8	0.1435	0.1440	-0.0005000	0.004014	8
Week 4 vs. Week 6	0.1449	0.1444	0.0005000	0.004014	8
Week 4 vs. Week 8	0.1449	0.1440	0.0008750	0.004014	8
Week 6 vs. Week 8	0.1444	0.1440	0.0003750	0.004014	8

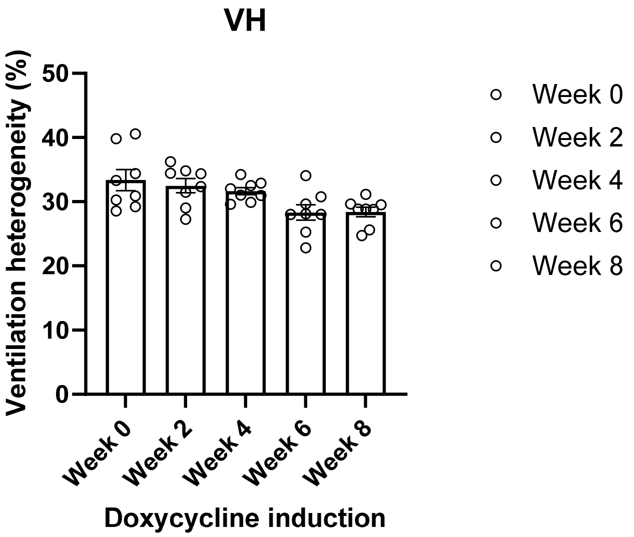

Tukey's multiple comparisons test	Mean Diff.	95.00% CI of diff.	Below threshold?	Summary	Adjusted P Value
Week 0 vs. Week 2	0.8888	-3.641 to 5.418	No	ns	0.9794
Week 0 vs. Week 4	1.738	-2.792 to 6.267	No	ns	0.8039
Week 0 vs. Week 6	5.053	0.5230 to 9.582	Yes	*	0.0225
Week 0 vs. Week 8	4.988	0.4580 to 9.517	Yes	*	0.0249
Week 2 vs. Week 4	0.8487	-3.681 to 5.378	No	ns	0.9826
Week 2 vs. Week 6	4.164	-0.3658 to 8.693	No	ns	0.0841
Week 2 vs. Week 8	4.099	-0.4308 to 8.628	No	ns	0.0919
Week 4 vs. Week 6	3.315	-1.215 to 7.845	No	ns	0.2411
Week 4 vs. Week 8	3.250	-1.280 to 7.780	No	ns	0.2587
Week 6 vs. Week 8	-0.06500	-4.595 to 4.465	No	ns	>0.9999
Test details	Mean 1	Mean 2	Mean Diff.	SE of diff.	n1
Week 0 vs. Week 2	33.38	32.49	0.8888	1.575	8
Week 0 vs. Week 4	33.38	31.65	1.738	1.575	8
Week 0 vs. Week 6	33.38	28.33	5.053	1.575	8
Week 0 vs. Week 8	33.38	28.40	4.988	1.575	8
Week 2 vs. Week 4	32.49	31.65	0.8487	1.575	8
Week 2 vs. Week 6	32.49	28.33	4.164	1.575	8
Week 2 vs. Week 8	32.49	28.40	4.099	1.575	8
Week 4 vs. Week 6	31.65	28.33	3.315	1.575	8
Week 4 vs. Week 8	31.65	28.40	3.250	1.575	8
Week 6 vs. Week 8	28.33	28.40	-0.06500	1.575	8

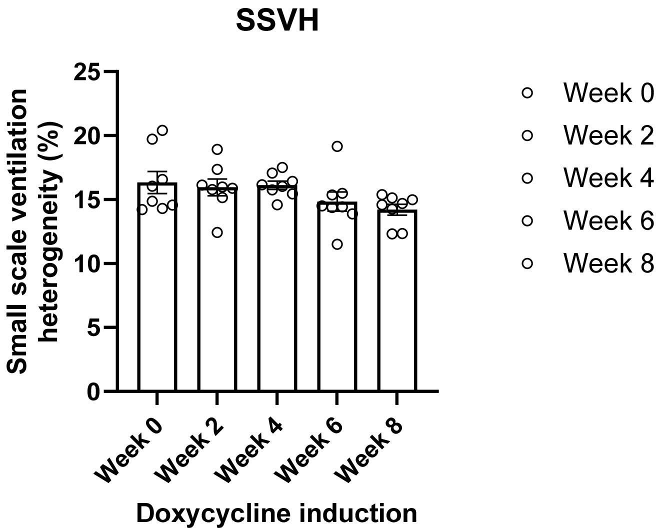

Tukey's multiple comparisons test	Mean Diff.	95.00% CI of diff.	Below threshold?	Summary	Adjusted P Value
Week 0 vs. Week 2	0.3775	-2.218 to 2.973	No	ns	0.9933
Week 0 vs. Week 4	0.2125	-2.383 to 2.808	No	ns	0.9993
Week 0 vs. Week 6	1.495	-1.101 to 4.091	No	ns	0.4734
Week 0 vs. Week 8	2.121	-0.4747 to 4.717	No	ns	0.1538
Week 2 vs. Week 4	-0.1650	-2.761 to 2.431	No	ns	0.9997
Week 2 vs. Week 6	1.118	-1.478 to 3.713	No	ns	0.7298
Week 2 vs. Week 8	1.744	-0.8522 to 4.340	No	ns	0.3204
Week 4 vs. Week 6	1.283	-1.313 to 3.878	No	ns	0.6190
Week 4 vs. Week 8	1.909	-0.6872 to 4.505	No	ns	0.2370
Week 6 vs. Week 8	0.6263	-1.970 to 3.222	No	ns	0.9566
Test details	Mean 1	Mean 2	Mean Diff.	SE of diff.	n1
Week 0 vs. Week 2	16.34	15.96	0.3775	0.9029	8
Week 0 vs. Week 4	16.34	16.13	0.2125	0.9029	8
Week 0 vs. Week 6	16.34	14.84	1.495	0.9029	8
Week 0 vs. Week 8	16.34	14.22	2.121	0.9029	8
Week 2 vs. Week 4	15.96	16.13	-0.1650	0.9029	8
Week 2 vs. Week 6	15.96	14.84	1.118	0.9029	8
Week 2 vs. Week 8	15.96	14.22	1.744	0.9029	8
Week 4 vs. Week 6	16.13	14.84	1.283	0.9029	8
Week 4 vs. Week 8	16.13	14.22	1.909	0.9029	8
Week 6 vs. Week 8	14.84	14.22	0.6263	0.9029	8

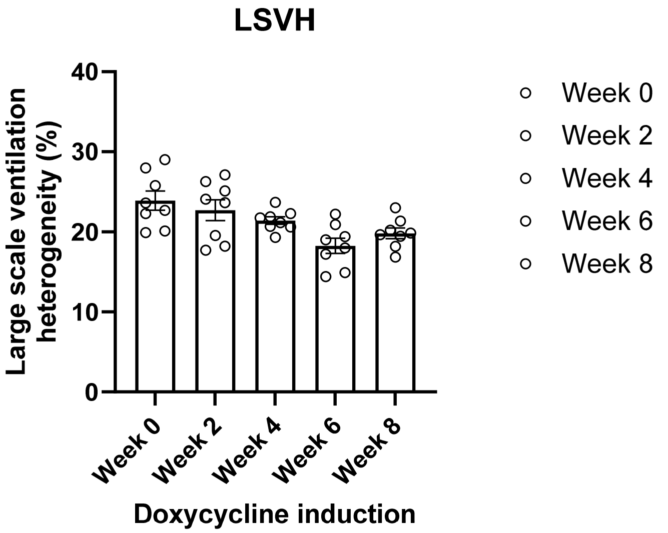

Tukey's multiple comparisons test	Mean Diff.	95.00% CI of diff.	Below threshold?	Summary	Adjusted P Value
Week 0 vs. Week 2	1.206	-2.745 to 5.158	No	ns	0.9032
Week 0 vs. Week 4	2.499	-1.453 to 6.450	No	ns	0.3797
Week 0 vs. Week 6	5.655	1.704 to 9.606	Yes	**	0.0020
Week 0 vs. Week 8	4.105	0.1537 to 8.056	Yes	*	0.0385
Week 2 vs. Week 4	1.293	-2.659 to 5.244	No	ns	0.8791
Week 2 vs. Week 6	4.449	0.4974 to 8.400	Yes	*	0.0208
Week 2 vs. Week 8	2.899	-1.053 to 6.850	No	ns	0.2390
Week 4 vs. Week 6	3.156	-0.7951 to 7.108	No	ns	0.1701
Week 4 vs. Week 8	1.606	-2.345 to 5.558	No	ns	0.7687
Week 6 vs. Week 8	-1.550	-5.501 to 2.401	No	ns	0.7908
Test details	Mean 1	Mean 2	Mean Diff.	SE of diff.	n1
Week 0 vs. Week 2	23.92	22.71	1.206	1.374	8
Week 0 vs. Week 4	23.92	21.42	2.499	1.374	8
Week 0 vs. Week 6	23.92	18.26	5.655	1.374	8
Week 0 vs. Week 8	23.92	19.81	4.105	1.374	8
Week 2 vs. Week 4	22.71	21.42	1.293	1.374	8
Week 2 vs. Week 6	22.71	18.26	4.449	1.374	8
Week 2 vs. Week 8	22.71	19.81	2.899	1.374	8
Week 4 vs. Week 6	21.42	18.26	3.156	1.374	8
Week 4 vs. Week 8	21.42	19.81	1.606	1.374	8
Week 6 vs. Week 8	18.26	19.81	-1.550	1.374	8

Figure S2: Statistical analysis of the longitudinal CCSP/- and CCSP/TGFα lung results. Graphical and tabular data derived from comparing changes between various time intervals post-doxycycline induction for each measured metric (BW, VDP, MSV, TV, VH, SSVH, and LSVH).

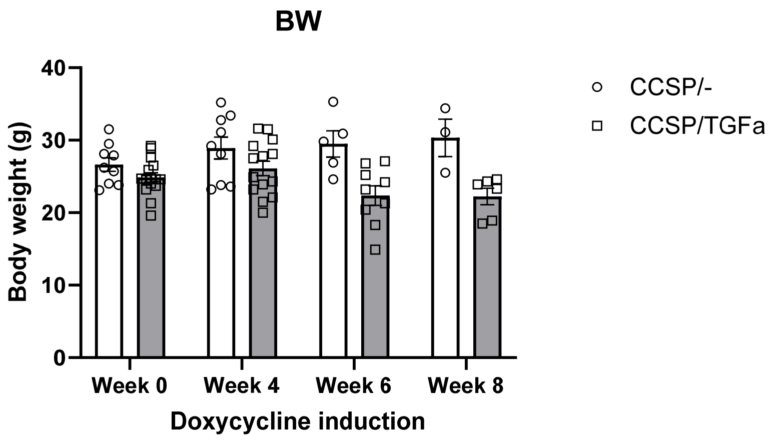

	Below threshold?	P value	Mean of CCSP/-	Mean of CCSP/TGFa	Difference	SE of difference
Week 0	No	0.127714	26.64	24.87	1.778	1.123
Week 4	No	0.123604	28.93	26.12	2.812	1.753
Week 6	Yes	0.008463	29.50	22.38	7.122	2.265
Week 8	Yes	0.011506	30.33	22.25	8.083	2.380

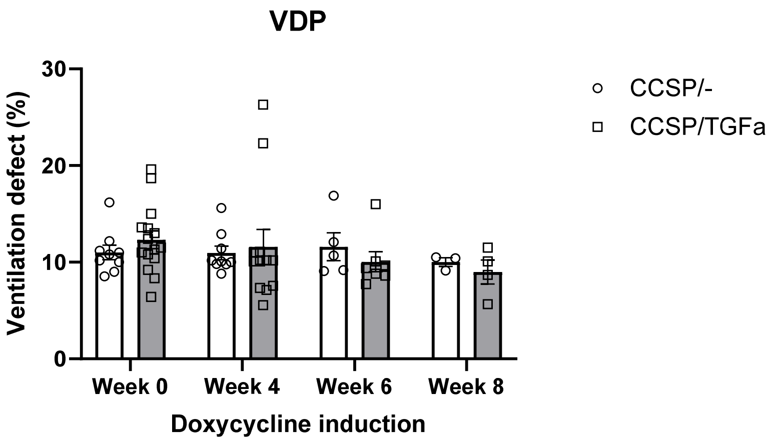

	Below threshold?	P value	Mean of CCSP/-	Mean of CCSP/TGFa	Difference	SE of difference
Week 0	No	0.334618	11.02	12.32	-1.299	1.317
Week 4	No	0.779320	10.96	11.58	-0.6203	2.182
Week 6	No	0.389476	11.59	10.05	1.547	1.719
Week 8	No	0.532612	10.01	8.990	1.020	1.523

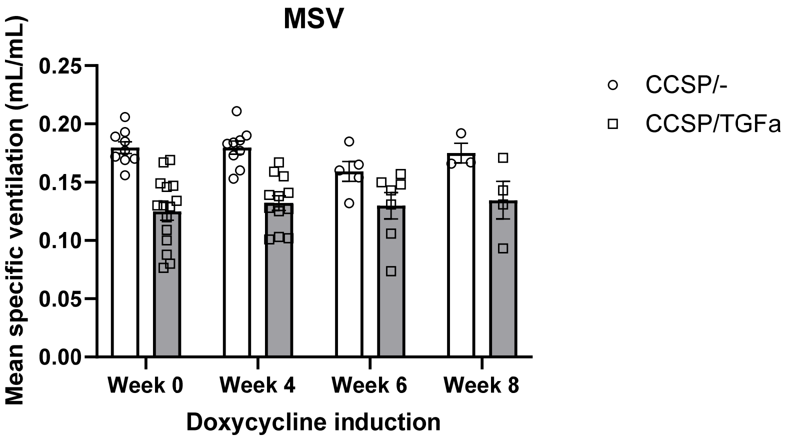

	Below threshold?	P value	Mean of CCSP/-	Mean of CCSP/TGFa	Difference	SE of difference
Week 0	Yes	0.000034	0.1797	0.1250	0.05470	0.01057
Week 4	Yes	0.000037	0.1797	0.1321	0.04758	0.008905
Week 6	No	0.083869	0.1592	0.1298	0.02939	0.01531
Week 8	No	0.103804	0.1750	0.1346	0.04045	0.02037

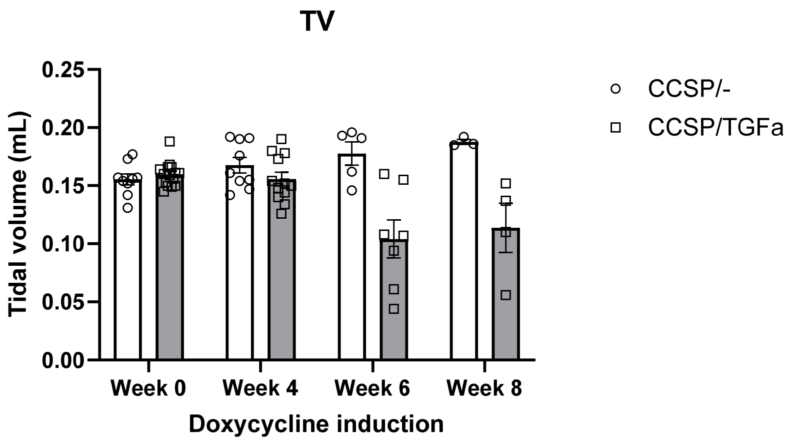

	Below threshold?	P value	Mean of CCSP/-	Mean of CCSP/TGFa	Difference	SE of difference
Week 0	No	0.402158	0.1554	0.1597	-0.004289	0.005020
Week 4	No	0.196223	0.1676	0.1558	0.01181	0.008814
Week 6	Yes	0.006343	0.1776	0.1041	0.07346	0.02136
Week 8	No	0.031886	0.1877	0.1138	0.07392	0.02506

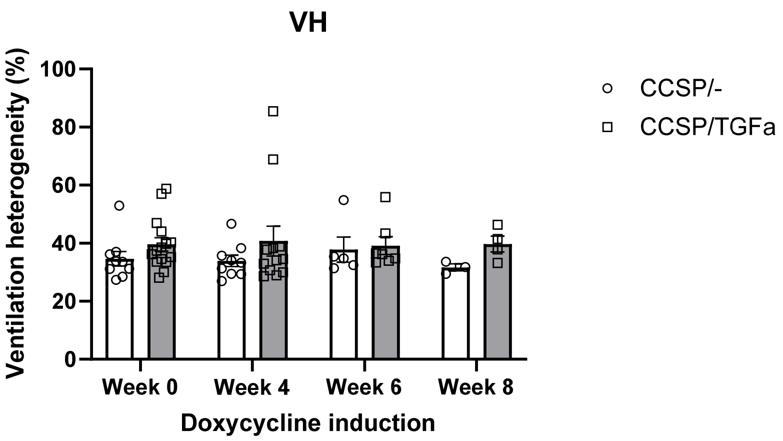

	Below threshold?	P value	Mean of CCSP/-	Mean of CCSP/TGFa	Difference	SE of difference
Week 0	No	0.175846	34.64	39.61	-4.974	3.556
Week 4	No	0.281336	33.91	40.76	-6.857	6.184
Week 6	No	0.794647	37.79	39.16	-1.372	5.132
Week 8	No	0.065852	31.65	39.72	-8.070	3.439

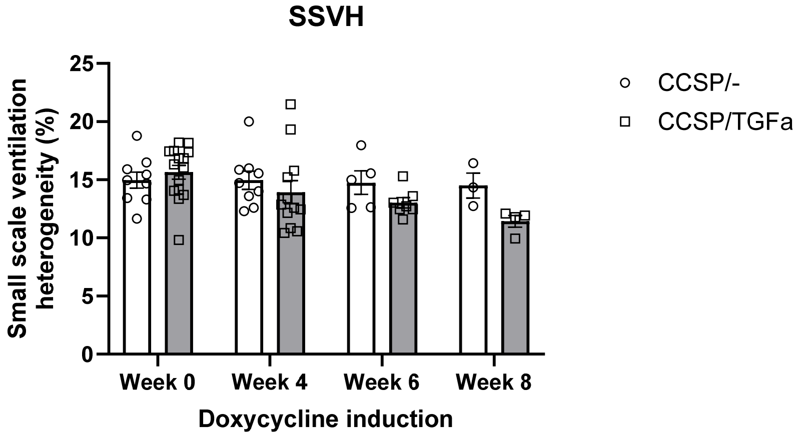

	Below threshold?	P value	Mean of CCSP/-	Mean of CCSP/TGFa	Difference	SE of difference
Week 0	No	0.468228	14.96	15.65	-0.6902	0.9351
Week 4	No	0.452305	14.96	13.93	1.030	1.342
Week 6	No	0.112082	14.75	13.02	1.722	0.9882
Week 8	No	0.035129	14.50	11.44	3.065	1.069

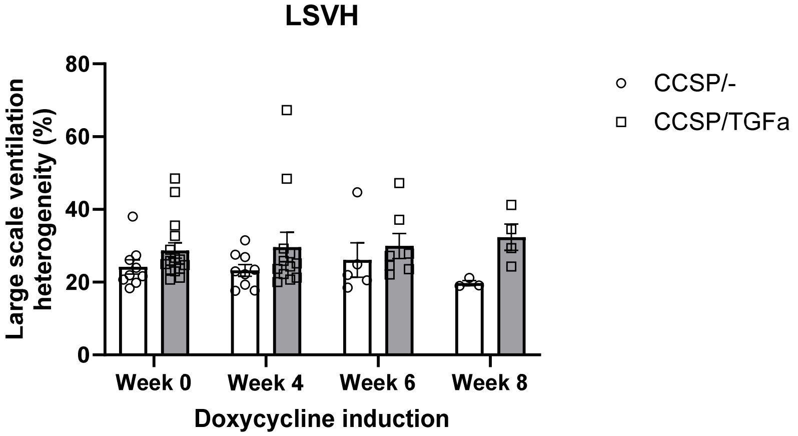

	Below threshold?	P value	Mean of CCSP/-	Mean of CCSP/TGFa	Difference	SE of difference
Week 0	No	0.169181	24.20	28.72	-4.520	3.180
Week 4	No	0.205677	23.23	29.66	-6.428	4.905
Week 6	No	0.514789	26.11	29.96	-3.850	5.701
Week 8	No	0.032828	19.76	32.37	-12.61	4.312

Figure S3: Effects of scanning protocols on imaging-derived functional lung metrics. Graphical and tabular data derived from comparing different scanning protocols for each measured functional lung metric (VDP, MSV, TV, VH, SSVH, and LSVH).

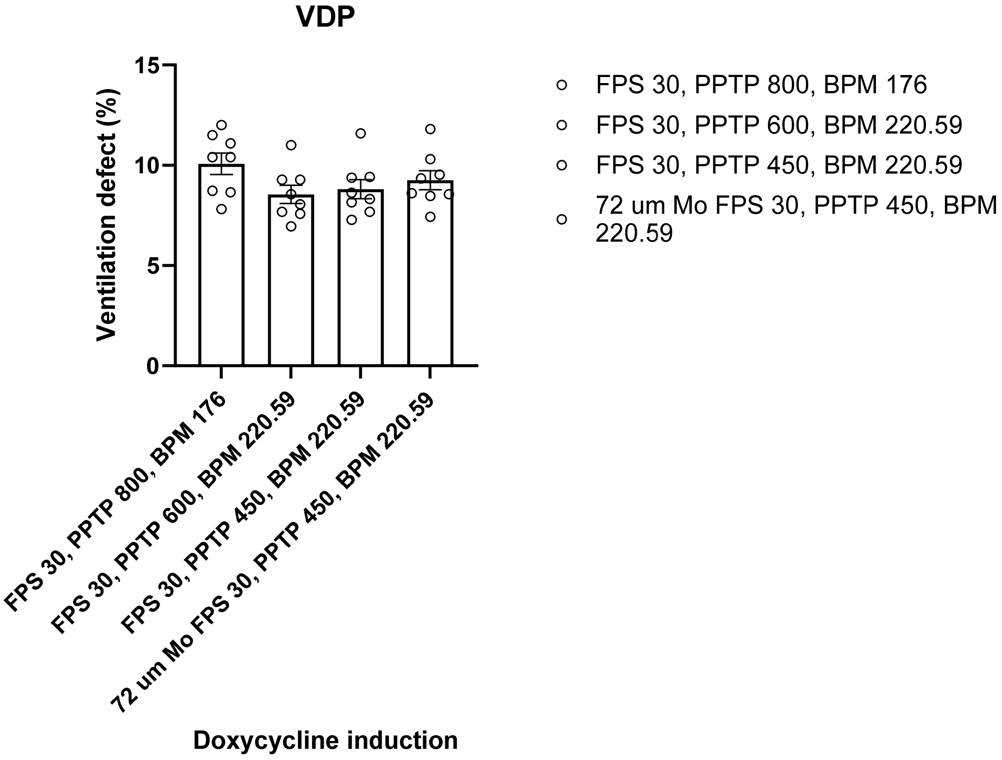

Tukey's multiple comparisons test	Mean Diff.	95.00% CI of diff.	Below threshold?	Summary	Adjusted P Value
FPS 30, PPTP 800, BPM 176 vs. FPS 30, PPTP 600, BPM 220.59	1.528	-0.3464 to 3.401	No	ns	0.1410
FPS 30, PPTP 800, BPM 176 vs. FPS 30, PPTP 450, BPM 220.59	1.266	-0.6077 to 3.140	No	ns	0.2744
FPS 30, PPTP 800, BPM 176 vs. Column D	0.8200	-1.054 to 2.694	No	ns	0.6351
FPS 30, PPTP 600, BPM 220.59 vs. FPS 30, PPTP 450, BPM 220.59	-0.2613	-2.135 to 1.613	No	ns	0.9808
FPS 30, PPTP 600, BPM 220.59 vs. Column D	-0.7075	-2.581 to 1.166	No	ns	0.7330
FPS 30, PPTP 450, BPM 220.59 vs. Column D	-0.4463	-2.320 to 1.428	No	ns	0.9146
Test details	Mean 1	Mean 2	Mean Diff.	SE of diff.	n1
FPS 30, PPTP 800, BPM 176 vs. FPS 30, PPTP 600, BPM 220.59	10.08	8.548	1.528	0.6863	8
FPS 30, PPTP 800, BPM 176 vs. FPS 30, PPTP 450, BPM 220.59	10.08	8.809	1.266	0.6863	8
FPS 30, PPTP 800, BPM 176 vs. Column D	10.08	9.255	0.8200	0.6863	8
FPS 30, PPTP 600, BPM 220.59 vs. FPS 30, PPTP 450, BPM 220.59	8.548	8.809	-0.2613	0.6863	8
FPS 30, PPTP 600, BPM 220.59 vs. Column D	8.548	9.255	-0.7075	0.6863	8
FPS 30, PPTP 450, BPM 220.59 vs. Column D	8.809	9.255	-0.4463	0.6863	8

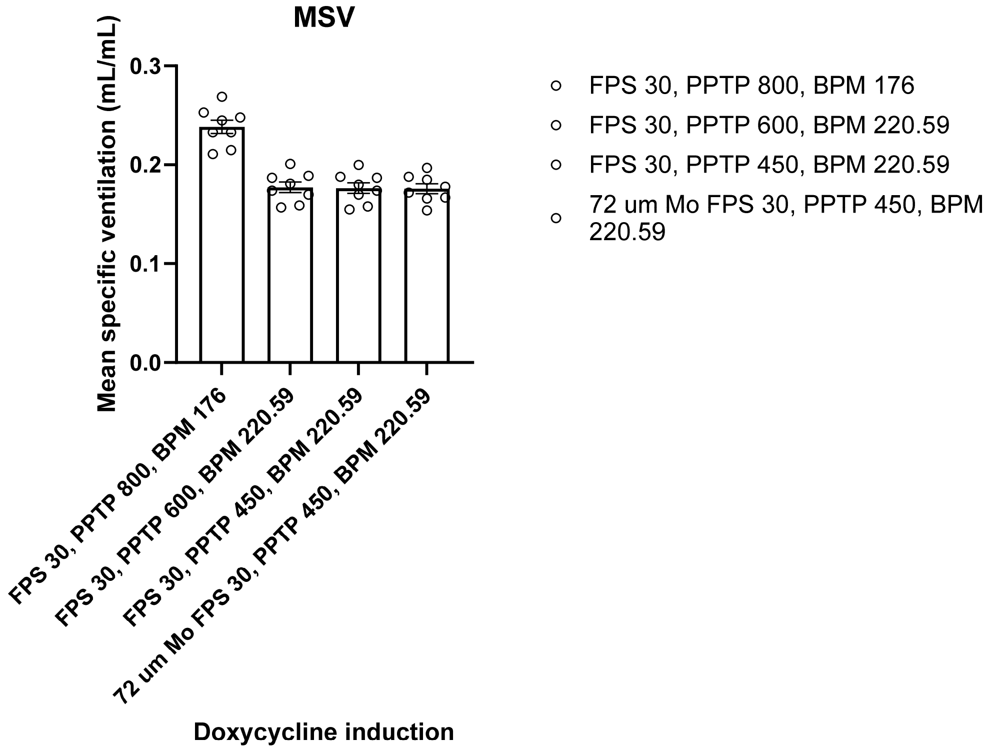

Tukey's multiple comparisons test	Mean Diff.	95.00% CI of diff.	Below threshold?	Summary	Adjusted P Value
FPS 30, PPTP 800, BPM 176 vs. FPS 30, PPTP 600, BPM 220.59	0.06113	0.03913 to 0.08312	Yes	****	<0.0001
FPS 30, PPTP 800, BPM 176 vs. FPS 30, PPTP 450, BPM 220.59	0.06188	0.03988 to 0.08387	Yes	****	<0.0001
FPS 30, PPTP 800, BPM 176 vs. Column D	0.06250	0.04050 to 0.08450	Yes	****	<0.0001
FPS 30, PPTP 600, BPM 220.59 vs. FPS 30, PPTP 450, BPM 220.59	0.0007500	-0.02125 to 0.02275	No	ns	0.9997
FPS 30, PPTP 600, BPM 220.59 vs. Column D	0.001375	-0.02062 to 0.02337	No	ns	0.9982
FPS 30, PPTP 450, BPM 220.59 vs. Column D	0.0006250	-0.02137 to 0.02262	No	ns	0.9998
Test details	Mean 1	Mean 2	Mean Diff.	SE of diff.	n1
FPS 30, PPTP 800, BPM 176 vs. FPS 30, PPTP 600, BPM 220.59	0.2384	0.1773	0.06113	0.008057	8
FPS 30, PPTP 800, BPM 176 vs. FPS 30, PPTP 450, BPM 220.59	0.2384	0.1765	0.06188	0.008057	8
FPS 30, PPTP 800, BPM 176 vs. Column D	0.2384	0.1759	0.06250	0.008057	8
FPS 30, PPTP 600, BPM 220.59 vs. FPS 30, PPTP 450, BPM 220.59	0.1773	0.1765	0.0007500	0.008057	8
FPS 30, PPTP 600, BPM 220.59 vs. Column D	0.1773	0.1759	0.001375	0.008057	8
FPS 30, PPTP 450, BPM 220.59 vs. Column D	0.1765	0.1759	0.0006250	0.008057	8

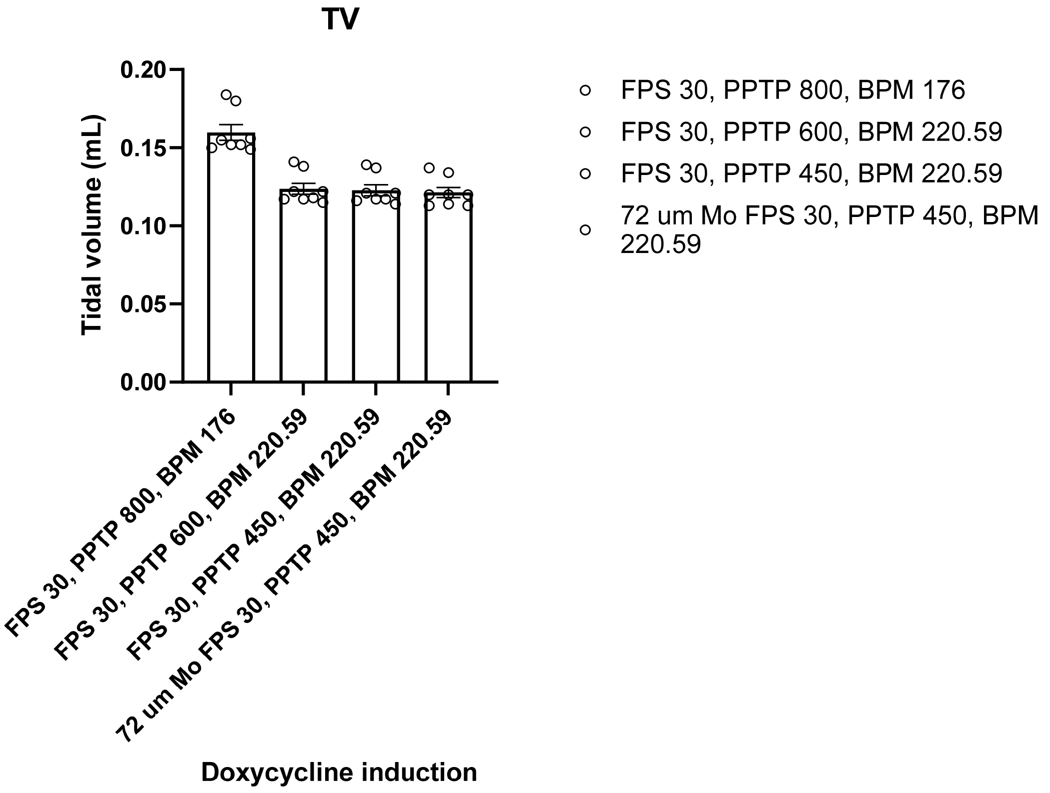

Tukey's multiple comparisons test	Mean Diff.	95.00% CI of diff.	Below threshold?	Summary	Adjusted P Value
FPS 30, PPTP 800, BPM 176 vs. FPS 30, PPTP 600, BPM 220.59	0.03600	0.02109 to 0.05091	Yes	****	<0.0001
FPS 30, PPTP 800, BPM 176 vs. FPS 30, PPTP 450, BPM 220.59	0.03700	0.02209 to 0.05191	Yes	****	<0.0001
FPS 30, PPTP 800, BPM 176 vs. Column D	0.03838	0.02347 to 0.05328	Yes	****	<0.0001
FPS 30, PPTP 600, BPM 220.59 vs. FPS 30, PPTP 450, BPM 220.59	0.001000	-0.01391 to 0.01591	No	ns	0.9978
FPS 30, PPTP 600, BPM 220.59 vs. Column D	0.002375	-0.01253 to 0.01728	No	ns	0.9719
FPS 30, PPTP 450, BPM 220.59 vs. Column D	0.001375	-0.01353 to 0.01628	No	ns	0.9943
Test details	Mean 1	Mean 2	Mean Diff.	SE of diff.	n1
FPS 30, PPTP 800, BPM 176 vs. FPS 30, PPTP 600, BPM 220.59	0.1598	0.1238	0.03600	0.005460	8
FPS 30, PPTP 800, BPM 176 vs. FPS 30, PPTP 450, BPM 220.59	0.1598	0.1228	0.03700	0.005460	8
FPS 30, PPTP 800, BPM 176 vs. Column D	0.1598	0.1214	0.03838	0.005460	8
FPS 30, PPTP 600, BPM 220.59 vs. FPS 30, PPTP 450, BPM 220.59	0.1238	0.1228	0.001000	0.005460	8
FPS 30, PPTP 600, BPM 220.59 vs. Column D	0.1238	0.1214	0.002375	0.005460	8
FPS 30, PPTP 450, BPM 220.59 vs. Column D	0.1228	0.1214	0.001375	0.005460	8

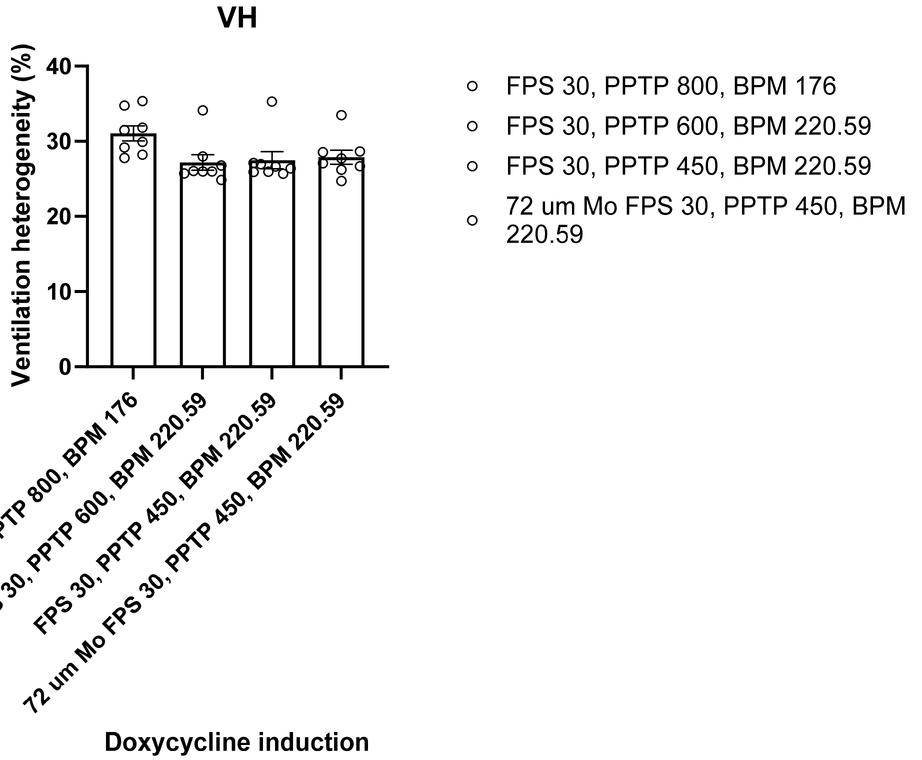

Tukey's multiple comparisons test	Mean Diff.	95.00% CI of diff.	Below threshold?	Summary	Adjusted P Value
FPS 30, PPTP 800, BPM 176 vs. FPS 30, PPTP 600, BPM 220.59	3.874	-0.08516 to 7.833	No	ns	0.0568
FPS 30, PPTP 800, BPM 176 vs. FPS 30, PPTP 450, BPM 220.59	3.568	-0.3914 to 7.526	No	ns	0.0887
FPS 30, PPTP 800, BPM 176 vs. Column D	3.171	-0.7877 to 7.130	No	ns	0.1516
FPS 30, PPTP 600, BPM 220.59 vs. FPS 30, PPTP 450, BPM 220.59	-0.3062	-4.265 to 3.653	No	ns	0.9966
FPS 30, PPTP 600, BPM 220.59 vs. Column D	-0.7025	-4.661 to 3.256	No	ns	0.9619
FPS 30, PPTP 450, BPM 220.59 vs. Column D	-0.3963	-4.355 to 3.563	No	ns	0.9927
Test details	Mean 1	Mean 2	Mean Diff.	SE of diff.	n1
FPS 30, PPTP 800, BPM 176 vs. FPS 30, PPTP 600, BPM 220.59	31.05	27.18	3.874	1.450	8
FPS 30, PPTP 800, BPM 176 vs. FPS 30, PPTP 450, BPM 220.59	31.05	27.49	3.568	1.450	8
FPS 30, PPTP 800, BPM 176 vs. Column D	31.05	27.88	3.171	1.450	8
FPS 30, PPTP 600, BPM 220.59 vs. FPS 30, PPTP 450, BPM 220.59	27.18	27.49	-0.3062	1.450	8
FPS 30, PPTP 600, BPM 220.59 vs. Column D	27.18	27.88	-0.7025	1.450	8
FPS 30, PPTP 450, BPM 220.59 vs. Column D	27.49	27.88	-0.3963	1.450	8

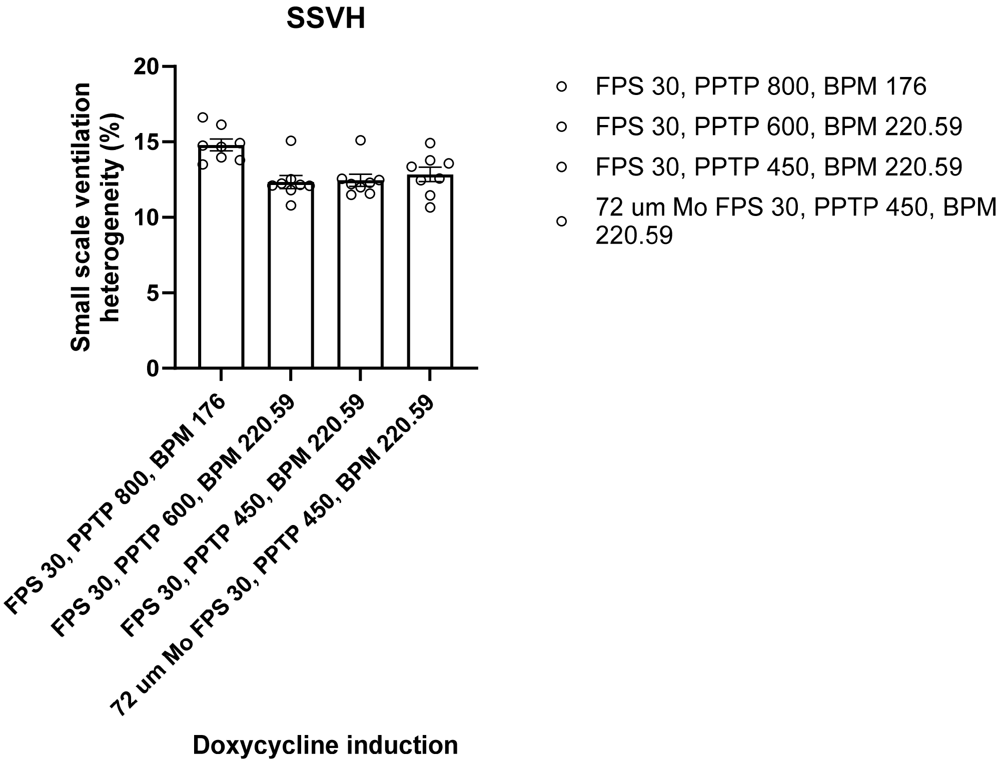

Tukey's multiple comparisons test	Mean Diff.	95.00% CI of diff.	Below threshold?	Summary	Adjusted P Value
FPS 30, PPTP 800, BPM 176 vs. FPS 30, PPTP 600, BPM 220.59	2.453	0.8038 to 4.101	Yes	**	0.0019
FPS 30, PPTP 800, BPM 176 vs. FPS 30, PPTP 450, BPM 220.59	2.336	0.6875 to 3.985	Yes	**	0.0032
FPS 30, PPTP 800, BPM 176 vs. Column D	1.953	0.3038 to 3.601	Yes	*	0.0156
FPS 30, PPTP 600, BPM 220.59 vs. FPS 30, PPTP 450, BPM 220.59	-0.1163	-1.765 to 1.532	No	ns	0.9974
FPS 30, PPTP 600, BPM 220.59 vs. Column D	-0.5000	-2.149 to 1.149	No	ns	0.8407
FPS 30, PPTP 450, BPM 220.59 vs. Column D	-0.3837	-2.032 to 1.265	No	ns	0.9197
Test details	Mean 1	Mean 2	Mean Diff.	SE of diff.	n1
FPS 30, PPTP 800, BPM 176 vs. FPS 30, PPTP 600, BPM 220.59	14.80	12.35	2.453	0.6039	8
FPS 30, PPTP 800, BPM 176 vs. FPS 30, PPTP 450, BPM 220.59	14.80	12.46	2.336	0.6039	8
FPS 30, PPTP 800, BPM 176 vs. Column D	14.80	12.85	1.953	0.6039	8
FPS 30, PPTP 600, BPM 220.59 vs. FPS 30, PPTP 450, BPM 220.59	12.35	12.46	-0.1163	0.6039	8
FPS 30, PPTP 600, BPM 220.59 vs. Column D	12.35	12.85	-0.5000	0.6039	8
FPS 30, PPTP 450, BPM 220.59 vs. Column D	12.46	12.85	-0.3837	0.6039	8

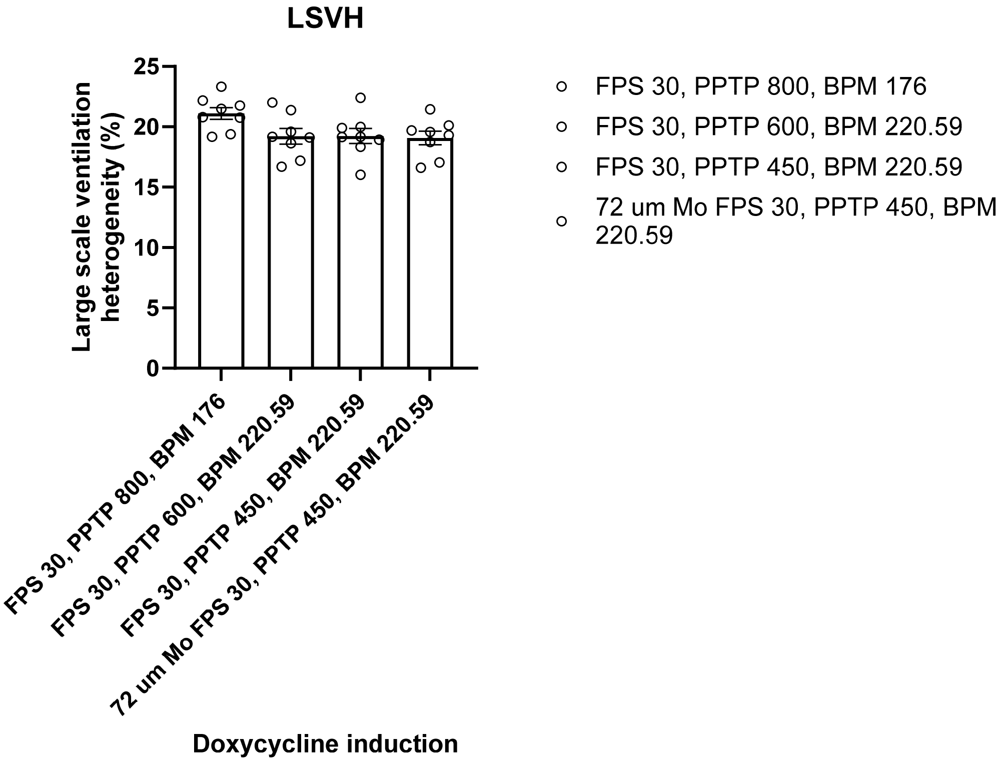

Tukey's multiple comparisons test	Mean Diff.	95.00% CI of diff.	Below threshold?	Summary	Adjusted P Value
FPS 30, PPTP 800, BPM 176 vs. FPS 30, PPTP 600, BPM 220.59	1.881	-0.3797 to 4.142	No	ns	0.1291
FPS 30, PPTP 800, BPM 176 vs. FPS 30, PPTP 450, BPM 220.59	1.864	-0.3972 to 4.125	No	ns	0.1344
FPS 30, PPTP 800, BPM 176 vs. Column D	2.039	-0.2222 to 4.300	No	ns	0.0884
FPS 30, PPTP 600, BPM 220.59 vs. FPS 30, PPTP 450, BPM 220.59	-0.01750	-2.278 to 2.243	No	ns	>0.9999
FPS 30, PPTP 600, BPM 220.59 vs. Column D	0.1575	-2.103 to 2.418	No	ns	0.9975
FPS 30, PPTP 450, BPM 220.59 vs. Column D	0.1750	-2.086 to 2.436	No	ns	0.9966
Test details	Mean 1	Mean 2	Mean Diff.	SE of diff.	n1
FPS 30, PPTP 800, BPM 176 vs. FPS 30, PPTP 600, BPM 220.59	21.11	19.23	1.881	0.8281	8
FPS 30, PPTP 800, BPM 176 vs. FPS 30, PPTP 450, BPM 220.59	21.11	19.24	1.864	0.8281	8
FPS 30, PPTP 800, BPM 176 vs. Column D	21.11	19.07	2.039	0.8281	8
FPS 30, PPTP 600, BPM 220.59 vs. FPS 30, PPTP 450, BPM 220.59	19.23	19.24	-0.01750	0.8281	8
FPS 30, PPTP 600, BPM 220.59 vs. Column D	19.23	19.07	0.1575	0.8281	8
FPS 30, PPTP 450, BPM 220.59 vs. Column D	19.24	19.07	0.1750	0.8281	8
